# Historically rice-farming societies have tighter social norms in China and worldwide

**DOI:** 10.1073/pnas.1909909117

**Published:** 2020-07-30

**Authors:** Thomas Talhelm, Alexander S. English

**Affiliations:** ^a^Booth School of Business, University of Chicago, Chicago, IL 60616;; ^b^Shanghai Intercultural Institute, Shanghai International Studies University, Shanghai 200083, China

**Keywords:** culture, norms, rice theory, subsistence style, tightness–looseness

## Abstract

Data recently published in PNAS mapped out regional differences in the tightness of social norms across China [R. Y. J. Chua, K. G. Huang, M. Jin, *Proc. Natl. Acad. Sci. U.S.A. *116, 6720–6725 (2019)]. Norms were tighter in developed, urbanized areas and weaker in rural areas. We tested whether historical paddy rice farming has left a legacy on social norms in modern China. Premodern rice farming could plausibly create strong social norms because paddy rice relied on irrigation networks. Rice farmers coordinated their water use and kept track of each person’s labor contributions. Rice villages also established strong norms of reciprocity to cope with labor demands that were twice as high as dryland crops like wheat. In line with this theory, China’s historically rice-farming areas had tighter social norms than wheat-farming areas, even beyond differences in development and urbanization. Rice–wheat differences were just as large among people in 10 neighboring provinces (*n* = 3,835) along the rice–wheat border. These neighboring provinces differ sharply in rice and wheat, but little in latitude, temperature, and other potential confounding variables. Outside of China, rice farming predicted norm tightness in 32 countries around the world. Finally, people in rice-farming areas scored lower on innovative thinking, which tends to be lower in societies with tight norms. This natural test case within China might explain why East Asia—historically reliant on rice farming—has tighter social norms than the wheat-farming West.

## Significance

Rice is a highly interdependent crop. Rice required far more labor than dryland crops like wheat, and rice’s irrigation networks forced farmers to coordinate water use. To deal with these demands, rice villages developed strong norms for labor exchange. Using China as a natural test case, we compare nearby provinces that differ in rice and wheat, but share the same ethnicity, religion, and national government. In survey data from over 11,000 Chinese citizens, rice-farming provinces report tighter norms than traditionally wheat-growing provinces. Rice also predicts tight norms around the world. These data suggest that China’s agricultural past still shapes cultural differences in the modern day—and perhaps explain why East Asia has tighter social norms than the wheat-growing West.

Social norms give structure to human societies—what it’s OK to do; what’s offensive; and what we’re morally obligated to do. All societies have norms, but when psychologists surveyed people in 32 nations around the world, they found that nations differed widely in the strength of those norms (1). In nations like India and South Korea, they found tight norms and low tolerance for deviation from accepted behavior. In nations like Israel and Venezuela, they found looser norms and more tolerance for nonconformists.

Tight norms seem to have consequences for people’s psychology. For example, people in societies with tight norms are better at self-monitoring, but also less creative (1–3). Here, we look not at the consequences of tight norms, but, rather, the question of where these norms come from. Researchers have found that norm differences across societies map onto factors like religion, urbanization, and disease (1–4). We ask whether histories of farming influence which cultures are tight and which are loose, even in places where farming is no longer a part of most people’s everyday life.

To get at this question, we leverage a natural test case. We compare regions in China that share the same national government, ethnicity, and language, yet differ in a variable that researchers have not tested as a cause of norm tightness—rice farming. First, we sketch out why the process of growing rice could plausibly leave a legacy of tight norms. We contrast rice with wheat, one of China’s other major historical grain crops. Although rice and wheat are both staples in China, we sketch out the reasons why wheat would allow for looser norms than rice.

We use China as a natural experiment, but the implications are not limited to China. If rice farming shapes culture, the consequences would extend to over half of the world’s population. Slightly more than 50% of the people in the world live in nations with a significant portion of wetland rice farming (5). Thus, after testing for differences in China, we test whether rice farming can explain norm tightness around the world.

## Norm Tightness in China

To measure norm tightness around China, we draw on a recent study in PNAS (2). In the study, researchers mapped out regional differences by surveying 11,662 people across China. They found stronger social norms in cities and highly developed provinces like Shanghai. Norms were weaker in less developed, rural provinces. For example, people in Shanghai were more likely than people from rural provinces to agree with statements like, “People agree upon what behaviors are appropriate versus inappropriate in most situations.”

Norms were also stronger in provinces that had experienced more threats. For example, norms were stronger in provinces that had higher rates of disease (table S5 in ref. 2), territorial occupation during World War II, and environmental disasters like chemical leaks and oil spills (ref. 2, p. 6721). In contrast, norms tended to be weaker in provinces that were more remote, farther from the central government in Beijing (ref. 2, p. 6721). Among all of the different factors, the authors flagged urbanization as a “key factor” in explaining which provinces had tighter norms.

## Why Rice Might Have Shaped Norms

China’s urban–rural divide is stark, but it is not the only cultural dividing line in China. The dividing line between rice and wheat cuts across the middle of China, near the Yangtze River. Around the Yangtze River and farther south, people have been farming rice for generations. Farther north, people have been farming dryland crops like wheat and millet.

Rice is a starkly different crop from wheat. Premodern paddy rice required twice the labor hours per hectare as crops like wheat, corn, and potatoes (6–8). Rice farming was more work because it involved tasks that wheat did not. For example, wheat farmers planted seeds directly in the field, but paddy rice farmers first planted seeds in small seedbeds (so that they could tightly control water levels), then later transplanted them to the field. What’s more, rice farmers completed these tasks in wet, muddy fields, which made even the same tasks take longer (7).

To deal with these demands, rice farmers exchanged labor. Far from a China phenomenon, labor exchange was common to rice around the world. Anthropologists have observed labor-exchange customs in rice villages from Japan to West Africa (7–9).

Of course, rice farmers are not the only people in the world who help each other. Wheat farmers help each other, too. Yet, the exchange is different. For example, one anthropologist compared labor exchange among dryland farmers in the Congo and rice farmers in Japan (10). Both groups exchanged labor, but the exchange of labor in rice areas was more critical and binding, whereas the exchange in the dryland areas was looser and more “festive” (10).

According to an anthropologist living in a Chinese rice village, if households “for any reason” can’t repay the labor they accept, they “must” hire workers to return the favor (11). Hiring laborers was expensive and inefficient, since paid laborers produced “careless work” and caused “wastage of grain” (11). The fact that farmers would use such a costly method signals just how strong norms were for labor exchange.

In short, rice villages had strong norms for reciprocity (7). If reciprocity really traces back to rice, it might be a factor that contributes to explaining modern-day differences in social ties across cultures. For example, asking people in Korea (a rice-farming culture) to think about how another person helped them triggered feelings of indebtedness, but it had no such effect on Americans (12).

## Rice Irrigation Relied on Norms

Another fundamental difference between rice and wheat is that paddy rice grows in standing water. By managing water levels, farmers can get yields four times larger than dryland rice (13). To get those yields, farmers needed irrigation systems. Those systems were not just engineering projects. They were social projects that profoundly shaped rice villages.

Contrast this with wheat villages, which often relied on rainfall. In those wheat villages, the weather coordinated the water. But when farmers came to control water, they had to start coordinating who got water. In some villages, the irrigation networks forced farmers to flood and drain their fields at the same time (8). If rice farmers disagreed about when to flood their fields, farmers who believed in early flooding would bicker with farmers who believed in later flooding. Ultimately, someone would have to win the argument, because the irrigation network gave them no choice.

Farmers linked together in irrigation systems also had to coordinate the work of dredging and repairing the channels (8, 9). It would have been difficult to coordinate that work in a society that had lots of people with their own ideas. An anthropologist in China found that everyone in the rice village he observed “can give, without hesitation” the arrangement of tasks from seed to harvest (11). Even a child recited “the complete annual agricultural cycle without omission or deviation from the proper order.” These shared norms make sense, as adaptations to help rice farmers coordinate their shared irrigation networks.

Beyond timing, farmers also needed to coordinate the labor needed to repair and dredge the irrigation channels every year (14). So, rather than allowing for individual differences, rice farmers set up systems to assign chores and monitor each other’s contributions. For example, rice farmers near Shanghai kept track of each person’s work assignments and punished people who failed to show up (9).

If farmers failed to fit in, punishments could cut deep. In Japan, rice farmers excluded uncooperative farmers from social life through *mura hachibu*, “80% separation from the village” (7). This legacy of strong norms might explain why people in modern-day Japan are more sensitive to social rejection than people in Western countries (15). It might also explain Japanese culture’s push to “not offend others,” as one cultural psychologist put it (16).

## Collectivism Does Not Always Require Coordination

Prior studies have found evidence that rice cultures are more collectivistic (5, 17) and that collectivistic societies tend to have tighter norms (*r* = 0.49, *P* = 0.01) (*SI Appendix*, Table S2 and Table 1). Thus, we could predict that rice cultures have tighter norms simply because they are more collectivistic. But this is a rather vague causal story. We argue that understanding the details of how people farmed rice produces more concrete reasons for how rice farming created tight norms.

**Table 1.** Rice-farming provinces have tighter social norms

	*B/γ*	SE	*t*	*P*
Rice				
Male	0.001	0.012	0.06	0.953
Age	0.001	0.001	1.90	0.058
GDP per capita	0.32	0.06	5.72	<0.001
% Urban	−0.76	0.23	−3.27	0.002
% Cultivated land	0.14	0.11	1.35	0.181
% Rice	0.12	0.04	2.90	0.005
Rice suitability				
Male	0.001	0.012	0.05	0.959
Age	0.001	0.001	1.89	0.058
GDP per capita	0.33	0.06	5.66	<0.001
% Urban	−0.69	0.23	−3.01	0.003
% Cultivated land	0.07	0.11	0.66	0.514
Environmental rice suitability	0.001	0.001	2.60	0.011
Rice–wheat border				
Male	0.01	0.02	0.43	0.666
Age	0.002	0.001	2.12	0.034
GDP per capita	0.33	0.13	2.62	0.016
% Urban	−1.04	0.60	−1.73	0.098
% Cultivated land	0.09	0.19	0.48	0.640
% Rice	0.21	0.09	2.20	0.040
Herding				
Male	0.001	0.012	0.06	0.951
Age	0.001	0.001	1.89	0.059
GDP per capita	0.32	0.07	4.50	<0.001
% Urban	−0.74	0.28	−2.67	0.009
% Cultivated land	0.16	0.14	1.16	0.249
% Rice	0.12	0.05	2.55	0.013
% Herding cultures	0.01	0.09	0.16	0.876

Analyses are hierarchical linear models with individuals nested in survey rounds nested in provinces. GDP is 2008 log Yuan. Urbanization is the percent of urban residents per province. Herding cultures is the square-root percent of the provincial population from traditionally herding cultures. The rice–wheat border analysis tests the percent rice among 10 neighboring provinces along China’s rice–wheat border.

Unpacking the specifics of rice farming can also be illuminating because it reveals what rice is *not*. Collectivism is a big concept. It encompasses multiple subfacets. For example, researchers have identified subfacets such as harmony, relying on other people, and being flexible to the situation (as opposed to being consistent across situations; ref. 18).

Yet rice farming would more plausibly cause some of these traits than others. For example, one study found that people from rice-farming areas of China were more likely than people from wheat areas to suspect that other people—even coworkers and classmates—were secretly trying to undermine them (19). Rice farming involves collectivism, but we predict that it does *not* require feeling warm, loving feelings toward others (7).

Similarly, interdependence does not always require coordination. One analogy is the difference social scientists have found between baseball and basketball (20, 21). Both sports make individual players interdependent. Single players can usually only win if their teammates do well. Even if baseball player Barry Bonds could hit a home run every time he came to the plate, he still wouldn’t win if his team's pitchers were terrible. Thus, both baseball and basketball players are interdependent in a way that tennis players and sprinters are not.

Yet, researchers have found evidence that baseball “depends far less on coordination” (21). Bonds hit those homeruns (mostly) by himself, without coordinating with his teammates. But as great a basketball player as Michael Jordan was, his scoring depended much more than Bonds’ on blocks and passes from his teammates. Thus, while both sports are interdependent, basketball requires more coordination. Coordination is clearly evident in traditional rice farming.

## Rice Versus Modernization

When testing for causes of norm tightness, we compared rice to causes that the previous study in China found—economic development and urbanization (2). We also went beyond the prior study by testing *historical* urbanization and economic development. This is important because research has found a lag between economic development and cultural change (22). Historical environments are sometimes a stronger predictor of cultural differences than current conditions (1, 23).

Finally, we expand on the prior study by testing indicators of economic modernization beyond gross domestic product (GDP). This is valuable because modernization theorists have argued that GDP is not the best marker of modernization (24, 25). Instead, researchers have argued that education or the shift to the modern service economy are better indicators of modernization (25). In China, the shift from the state-run economy to the private sector may be another important indicator of modernization. Thus, we analyze data on private industry, the service economy, and education (again, testing both modern and historical indicators).

## Does Urbanization Lead to Stronger Norms in China, But Not in the United States?

The results replicated the prior finding that social norms are tighter in more developed provinces (γ = 0.32, *P* < 0.001, *r*_prov_ = 0.59; γ represents group-level regression coefficients). Yet, we dug deeper on a surprising result from the prior study—that Chinese cities have tighter norms than rural areas. This is surprising because a study in the United States found that cities have *weaker* norms (3). Another reason it is surprising is because studies have found that people in cities tend to be more creative (26), which is more common in areas with loose norms (1). Cities also tend to be more individualistic (27), which, again, is associated with loose norms (1). If true, the prior finding raises the intriguing possibility that urbanization somehow works differently in China than other places (28).

But, after accounting for GDP, the paradoxical finding reversed (Table 1). Urbanization now predicted *less* strong norms (γ = −0.01, *P* = 0.002, *r*_prov_ = −0.37). When comparing places in China that are similarly wealthy, urbanized areas tend to have looser norms. Thus, the paradoxical finding seems to have been a confound of economic development.

## Rice Areas Have Tighter Social Norms

**Fig. 1.**
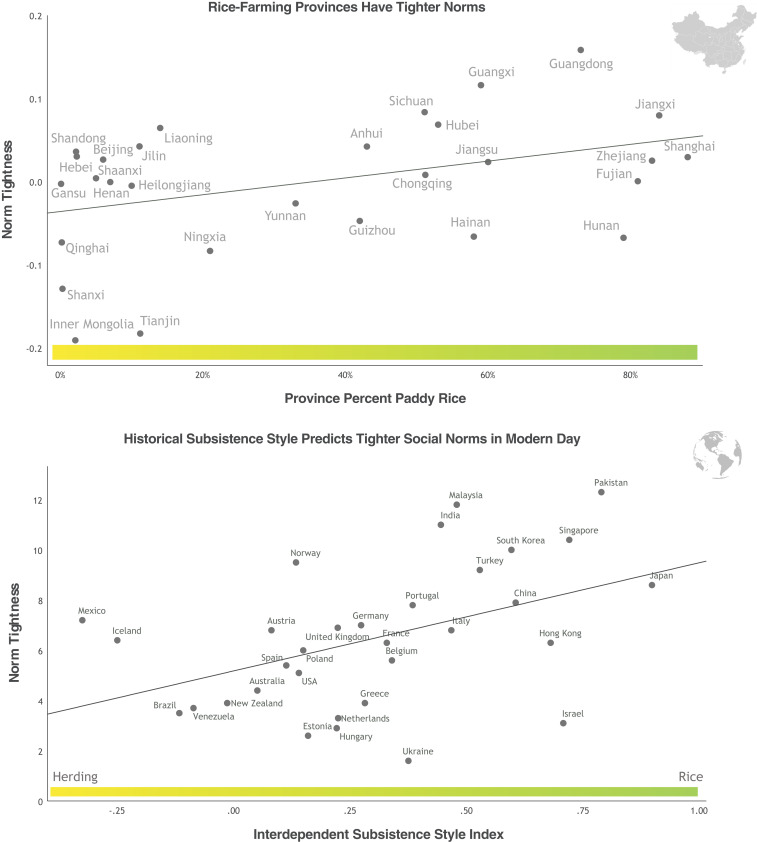
(*Upper*) Rice-farming provinces in China have tighter social norms. (*Lower*) Around the world, societies that practiced more interdependent subsistence styles had tighter norms. This index accounts for land devoted to wheat, herding (less interdependent), and rice (more interdependent). Province scores control for urbanization and GDP.

Rice-farming areas had tighter social norms in a simple analysis (γ = 0.10, *P* = 0.043, *r*_prov_ = 0.20) and after taking into account GDP and urbanization (γ = 0.12, *P* = 0.005, *r*_prov_ = 0.33; Fig. 1 and Table 1). Rice was robust to controlling for respondents’ age and education (Table 1 and *SI Appendix*, Table S6). Rice remained significant after taking into account the three different rounds of the survey, which stretched across 3 y.

Economic development explained the most variation in norm tightness (*r*_prov_ = 0.59). Rice (*r*_prov_ = 0.33) explained about as much variation as urbanization (*r*_prov_ = 0.37). In sum, both modern development and historical rice farming predicted patterns of norm strength across China.

## Rice Farming Is Key, Not Farming in General

One reasonable doubt is whether tight norms are specific to rice farming or farming in general. We pulled apart rice farming from farming in general by analyzing the percentage of cultivated land in different provinces (Table 1). Rice continued to predict tighter norms after taking into account farming in general. Thus, rice seems to have effects apart from farming in general.

## Rice Effect Is Separate from Population Density

Researchers have theorized that societies develop tighter norms in response to population pressure (1). The idea is that tight social norms help societies deal with the dangers of crowding, such as disease and poor sanitation. There is some evidence for this. Around the world, nations with denser populations have tighter social norms (1), although this is not true among US states (3).

In China, densely populated provinces have tighter norms (*r*[29] = 0.54, *P* = 0.002). But this correlation should be treated with caution because population density is highly correlated with urbanization (*r*[29] = 0.71, *P* < 0.001) and GDP (*r*[29] = 0.68, *P* < 0.001) in China. When we pitted all three factors against each other in a single model, population density was no longer significant (*P* = 0.698), while GDP and urbanization remained highly significant (*SI Appendix*, Table S5). Even pitting just GDP and population density against each other left population density nonsignificant. Thus, population density was not a robust predictor of norms in China.

Another possibility is that historical population density has a stronger influence on culture. Across nations, historical population density is a stronger predictor of norms than modern population density, perhaps because it more precisely reflected societies’ long-run history (1). We tested this possibility in China using population-density estimates from the 1700s for 22 provinces (*SI Appendix*, Table S1). However, results were similar to modern density (*SI Appendix*, Table S5). Historical density was correlated with tight norms, but this relationship disappeared after controlling for GDP.

We also ran analyses to explore whether population density might operate as a mechanism between rice and social norms. This could make sense with the fact that rice was far more productive per hectare than wheat and so could support denser populations (7). However, rice was only modestly correlated with population density in China (*r*[29] = 0.36, *P* = 0.045).

Instead, population density was far more closely related to the density of farming in general (*r*[29] = 0.72, *P* < 0.001). Shandong province in northern China is a good example. Shandong is a wheat province, yet it is one of China’s densest farming areas, with 42% of land devoted to farming. In contrast, Guangdong and Fujian are rice provinces, but with only about 10% of land devoted to farming.

The fact that rice and population density are separable can explain why rice remained significant after controlling for population density, both modern and historical (*SI Appendix*, Table S5). Thus, rice–wheat differences seem to be operating outside of population density. However, we caution that provinces are coarse units of analysis. With such coarse units, it's hard to pull apart rice and population density. If future studies can gather county-level tightness data, they will have better granularity to test these factors.

## Herding

Another plausible historical factor that could have influenced norm strength across China is herding. Herding cultures tend to be more individualistic than farming cultures (29, 30), and individualistic cultures tend to have looser norms (1). In line with this idea, a recent worldwide study found that herding cultures tend to have looser, more flexible social relationships (23). However, herding areas in China did not have looser norms (Table 1).

## Environmental Threats

One potential explanation for why herding areas did *not* have looser norms is that China’s herding areas also experienced more historical warfare (Table 2). This is important because norms tend to be tighter in places that have experienced war and other types of environmental threats (1, 2). We tested this theory using historical data on disease prevalence, the frequency of war, and mass uprisings across China (Table 2 and *SI Appendix*, Table S4). Based on a simple correlation, regions that experienced more warfare had marginally tighter norms (*r*[29] = 0.36, *P* = 0.050). However, warfare was more common in wealthier provinces, and warfare became nonsignificant after controlling for GDP (Table 2).

**Table 2.** Rice farming is robust to historical rebellion, warfare, and area occupied by Japan in WWII

	*B/γ*	SE	*t*	*P*
Historical rebellion				
Male	0.0004	0.0124	0.03	0.973
Age	0.001	0.001	1.91	0.056
GDP per capita	0.31	0.06	5.56	<0.001
% Urban	−0.64	0.24	−2.64	0.010
% Cultivated land	0.23	0.11	1.98	0.051
% Rice	0.13	0.04	3.25	0.002
Historical rebellion	−0.04	0.02	−1.84	0.069
Historical warfare				
Male	0.001	0.012	0.05	0.960
Age	0.001	0.001	1.90	0.058
GDP per capita	0.30	0.07	4.52	<0.001
% Urban	−0.70	0.25	−2.85	0.005
% Cultivated land	0.18	0.12	1.54	0.127
% Rice	0.12	0.04	2.92	0.005
Historical warfare	0.004	0.005	0.76	0.448
WWII occupied area				
Male	0.001	0.013	0.09	0.925
Age	0.001	0.001	1.19	0.236
GDP per capita	0.33	0.06	5.58	<0.001
% Urban	−0.76	0.23	−3.27	0.002
% Cultivated land	−0.06	0.14	−0.43	0.670
% Rice	0.10	0.04	2.43	0.017
Area occupied by Japan WWII	0.06	0.04	1.32	0.191

Studies have found that areas with more history of warfare have tighter norms (1). Rebellion data are an index of the frequency mass rebellions during the Qing Dynasty (1644–1911, from ref. 30). “Historical warfare” is the number of battles in wars with an external foe in the Qing Dynasty (30). The proportion of provincial area occupied by Japan during WWII comes from Chua et al. (2). Occupied area significantly correlates with tightness (*r*[28] = 0.59, *P* = 0.001), but becomes nonsignificant when controlling for GDP per capita. This is because rich coastal provinces were occupied to a greater extent (*r*[28] = 0.62, *P* < 0.001). GDP data are log Yuan from 2008. Urbanization is the percentage of urban residents per province in 2017.

Rice continued to predict norm tightness after accounting for warfare, disease, and a series of alternative explanations and potential confound variables (*SI Appendix*, Tables S1 and S3–S9 describe all variables and theories tested). Rice was robust to distance from the coast (a proxy for trade and economic development), distance from Beijing (because norms tend to be tighter nearer to the central government; ref. 2), ethnic homogeneity, and excluding outlying provinces like Tibet.

We also tested a wider set of indicators of modernization, such as service-sector employment, private enterprise, and education (*SI Appendix*, Tables S6–S8). In each case, we tested both modern and historical indicators. In line with the idea that there is a lag between economic development and cultural change (22), we found that historical GDP predicted norms better than the modern GDP statistics used in the original study (*SI Appendix*, Table S8*C*).

## Differences Just as Large Along Rice–Wheat Border

One problem with using China as a natural test case is that rice is not randomly distributed. Instead, rice is highly correlated with temperature and latitude (|*r*|s ≥ 0.78). To get around this problem, we compared people from 10 neighboring provinces along the rice–wheat border (*n* = 3,835). The border gives a cleaner test case of areas that differ starkly in rice, but much less in temperature and other variables. For example, Jiangsu province farms 60% rice, while neighboring Shandong farms just 2% rice.

Norms differed significantly along the rice–wheat border (Table 1). Differences were as large along the rice–wheat border (*r*_prov_ = 0.43) as for China as a whole (*r*_prov_ = 0.33). This result suggests that rice–wheat differences are independent from other factors that differ between northern and southern China as a whole, such as temperature and contact with herding cultures.

## Environmental Suitability to Rice

If tight norms help people farm rice, it raises the question of reverse causality. Our theory is that rice causes tight norms, but the opposite could also be possible. Maybe people in China who *already had* tight norms *chose* to farm rice. One way to test whether people in some parts of China chose to farm rice is to ask where it’s physically possible to grow rice. If all of China could grow rice, but only the regions that have tight norms actually grow rice, this would suggest that tight norms caused people to farm rice (reverse causality).

To test this idea, we mapped out where it’s possible to grow rice using climate data from the United Nations Food and Agriculture Organization’s Global Agro-Ecological Zones Database. This database estimates the environmental suitability for wetland rice based on temperature, slope, soil, and other variables from 1961 to 1990. Environmental suitability strongly predicted actual rice farming across China (β = 0.87, *P* < 0.001). Environmental rice suitability also predicted tighter norms (γ = 0.001, *P* = 0.011, *r*_prov_ = 0.29) (Table 1).

This suggests that the environment determined where people farm rice in China. Rice spread to the provinces where it was ecologically possible, probably because it was five times more productive per hectare than wheat (7). Although this analysis cannot entirely rule out reverse causality, the results suggest that reverse causality is not likely to be driving these cultural differences.

## Rice May Be Behind East–West Differences

One intriguing possibility is that rice farming might help explain cultural differences beyond China. Theorists have proposed many different explanations for East–West differences, such as Confucianism and Eastern “despotism” (31, 32). We propose that East Asia’s history of rice farming has played at least some role in putting it on a different path from the wheat-farming West.

**Fig. 2.**
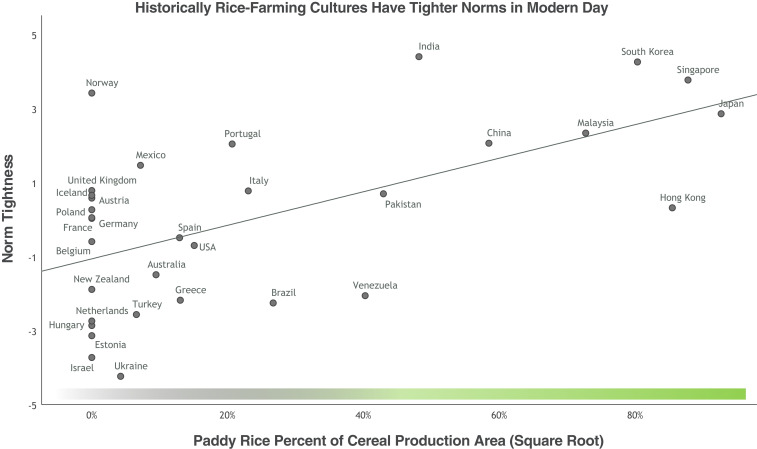
Rice farming and norm tightness around the world. Norm data come from a study by Gelfand et al. (1). Because Islamic countries tend to have stronger norms, and several rice-farming cultures (such as Pakistan) have a high percentage of Muslims, the graph controls for percentage of Muslims. Rice is significant whether Islam is controlled for or not (*SI Appendix*, section 20).

We investigated this idea by testing whether rice farming predicts differences outside of China. To do this, we analyzed the tightness–looseness data from 32 nations around the world from Gelfand et al. (1). We found that countries that devoted more cultivated land to paddy rice have tighter social norms (*r*[30] = 0.51, *P* = 0.003) (Fig. 2). Rice farming continued to predict tight norms after taking into account modern economic development, historical development, urbanization, and environmental threats (Table 2 and *SI Appendix*, Table S10). Although rice is linked to interdependence (17), rice continued to predict norm tightness across China and around the world, even after taking into account survey measures of individualism and collectivism (*SI Appendix*, Table S12). This could suggest that rice influences norm tightness through means other than interdependence or that the survey measures are not precisely measuring the interdependence of rice farming.

Yet rice is just one subsistence style. Herding is another common traditional subsistence style around the world, and herding cultures tend to be more independent, with looser relationships (23, 29). To index herding, we used data on land devoted to herding around the world (*SI Appendix*, section 4). The data showed that herding cultures had less tight norms (*r*[30] = −0.45, *P* = 0.010).

Finally, we combined rice farming, wheat farming, and herding into a broader index of subsistence styles (23). This index estimates the interdependence of different nations’ subsistence styles. The index takes the proportion of cereal land devoted to wheat minus herding land (less interdependent) plus rice land (more interdependent; *SI Appendix*, section 4 describes the index in detail). The interdependent subsistence-style index predicted norm tightness around the world (*r*[30] = 0.46, *P* = 0.009) (Fig. 1). Subsistence styles continued to predict norms after taking into account GDP, urbanization, and environmental threats (Table 3 and *SI Appendix*, Table S11).

**Table 3.** Societies with more rice farming and more interdependent subsistence styles have tighter norms

	*B*	SE	*t*	*P*	*B*	SE	*t*	*P*	*B*	SE	*t*	*P*	*B*	SE	*t*	*P*
Rice																
% Rice harvested area	5.69	1.75	3.24	0.003	6.52	1.50	4.35	<0.001	5.50	1.48	3.71	0.001	5.95	1.42	4.19	<0.001
GDP per capita (2011 $10,000 PPP)	−0.11	0.07	−0.40	0.693									0.56	0.29	1.96	0.060
% Urban					−8.22	2.48	−3.32	0.002					−9.13	3.58	−2.55	0.017
Historical and ecological threats									0.07	0.02	3.23	0.003	0.04	0.03	1.39	0.177
Subsistence style																
Interdependent subsistence style index	4.30	1.56	2.76	0.010	3.95	1.49	2.65	0.013	3.56	1.46	2.44	0.021	3.28	1.45	2.26	0.032
GDP per capita (2011 $10,000 PPP)	0.04	0.29	0.13	0.898									0.04	0.03	1.39	0.177
% Urban					−5.15	2.83	−1.82	0.078					−5.87	4.16	−1.41	0.170
Historical and ecological threats									0.06	0.03	2.36	0.025	0.55	0.34	1.61	0.118

Tightness–looseness values come from the Gelfand et al. (1) study of 32 nations. Rice is the percent of cereal-production area harvested with rice. The subsistence index combines rice farming, wheat farming, and herding. Nations with more rice farming score higher on interdependence, while nations with more herding score lower on interdependence. Threat data are an index of seven threats, such as disease, warfare, and natural disasters. Gelfand et al. (1) identified these types of threats, and a later study on relational mobility combined them into a single index (19). PPP, purchasing power parity.

## Rice Farming Linked to Thought Style

Next, we looked at data on one of the proposed consequences of tight norms. Tight cultures emphasize fitting in, which is good for coping with threats, but bad for creativity (1, 3, 33, 34). In China, provinces with tighter norms scored lower on a measure of innovative thinking style and had fewer patents for inventions (2). If rice encourages tight norms, does it also predict differences in thought style and innovation?

Using the thought-style data from Chua et al. (2), we found that rice-farming areas had lower innovative thinking style (γ = −0.11, *P* = 0.013, *r*_prov_ = −0.63) (*SI Appendix*, Fig. S1 and Table 4). Next, we asked whether rice farming is linked to lower innovation because of norm tightness. A mediation analysis revealed that norm tightness explained a portion of the relationship between rice and lower innovative thought (*B* = 0.043 [95% CI = 0.013; 0.072], *Z* = 2.81, *P* = 0.005; *SI Appendix*, section 1.1). In other words, rice-farming provinces have tighter norms, which are then linked to creativity (*SI Appendix*, Fig. S4). However, norm tightness only explained a portion of the relationship between rice and innovation. This suggests that there are pathways other than norm tightness at work.

**Table 4.** Rice farming predicts lower innovative thought style

	*B/γ*	SE	*t*	*P*
Innovation				
Male	0.10	0.02	5.51	<0.001
Age	−0.0003	0.0010	−0.33	0.743
GDP per capita	0.02	0.06	0.30	0.766
% Urban	0.03	0.24	0.11	0.916
% Cultivated land	−0.02	0. 11	−0.20	0.844
% Rice	−0.11	0.04	−2.69	0.013
Conformity				
Male	0.003	0.016	0.18	0.860
Age	0.001	0.001	1.56	0.118
GDP per capita	0.10	0.05	1.88	0.072
% Urban	−0.32	0.21	−1.48	0.152
% Cultivated land	−0.07	0.10	−0.72	0.477
% Rice	−0.03	0.04	−0.88	0.389
Efficiency				
Male	0.02	0.02	0.98	0.328
Age	0.007	0.001	7.43	<0.001
GDP per capita	0.09	0.06	1.47	0.154
% Urban	−0.13	0.26	−0.50	0.620
% Cultivated land	0.05	0.12	0.42	0.679
% Rice	−0.06	0.04	−1.29	0.209

Analyses are hierarchical linear models with individuals nested in survey rounds nested in provinces. GDP data are log Yuan from 2008. Urbanization is the percentage of urban residents per province in 2017. Thought-style data come from Chua et al. (2), using Kirton’s adaption–innovation inventory (29).

These results are consistent with the idea that rice farming encourages tight norms to deal with the high labor demands of rice, but that this tightness comes at the cost of innovative thinking. The finding of lower innovative thought style is consistent with two earlier studies that found lower rates of invention patents in China’s rice areas (17, 35). In sum, separate datasets provide converging evidence linking rice to thought style and innovation.

## Limitations

One important limitation with this analysis is that it compares provinces. Provinces are coarse units. With only 31 provinces, it is hard to pull different variables apart. For example, urbanization is highly correlated with GDP (*r*[29] = 0.89, *P* < 0.001). Future studies can pull these variables apart more finely by collecting county-level data.

There are also several unanswered questions. For example, what elements of rice farming can be abstracted out to predict tight norms in other types of groups? Understanding this would help us predict beyond the three major subsistence styles we test here. Humans have developed many ways of eating and surviving besides rice, wheat, and herding.

There are some hints about other subsistence styles in prior research. For example, a team of researchers ran economic games in 15 small-scale societies around the world (36). Using the classic Ultimatum Game, they found the most generous offers among the Lamalera people of Indonesia. The Lamalera hunt whales. Taking down a whale is probably an impossible task for a single person. Instead, the Lamalera work together in teams to bring down their large prey, then split the harvest with other people back on shore. Data from more small-scale societies like these can give us a more detailed picture of which types of subsistence styles tend to create tight norms.

## Conclusion

The earlier study found the paradoxical result that Chinese cities have tighter norms than rural areas (2). This contradicts data from the United States, where cities have looser norms (3). This is also surprising because cities are hubs of creativity (26) and individualism (27), which are both more common where norms are loose (1, 2).

Maybe cities work differently in China. A commentary on the earlier findings offered one plausible explanation (28). Cities in China are dense with cameras and monitoring, which could make people feel more pressure to follow the rules. Yet, when we compared places that were similarly wealthy, the more urbanized areas tended to have *looser* norms. Thus, the data support the idea that cities are generally loose, rather than a China-specific pattern.

The analysis also revealed evidence for a lag between economic change and cultural change. Studies of cultural differences often control for recent GDP statistics—even in studies that test for effects of historical factors. Yet, there is evidence of a lag time between economic development and cultural change (22, 23). In China, GDP data from a decade before the survey predicted more variation in norm tightness than current GDP data (*SI Appendix*, Table S8*C*). To us, these findings suggest that testing historical indicators should become an expected standard in cultural research.

### The Causes of Norm Tightness.

This study advances our understanding of cultural differences by testing potential causes of those differences (1–3). This can be difficult when comparing different nations. If we compare, say, the United States and China, factors like language, religion, and government are mixed together with factors like rice and wheat farming. The data here are particularly valuable because they leverage China as a natural test case. Comparing within China allows us to compare areas that share factors like language and national government, yet differ in rice and wheat.

Another way that this study (and other recent studies) on norm tightness have helped advance the field of cultural psychology is by pushing beyond individualism and collectivism. Individualism and collectivism are large, sometimes fuzzy concepts. Cultural psychologists have called for moving beyond collectivism to more precise traits (18). These studies on norm tightness are among a handful of studies in recent years that have pushed beyond collectivism (such as refs. 1, 23).

### Real-World Consequences.

These results have real-world implications for Chinese society. Studies have linked norm tightness to important real-world outcomes (28). Tightness seems to bring some benefits. For one, tight norms seem to be useful for social coordination. People in societies with tight norms prioritize social order, self-regulate more, and abuse drugs less (28).

But tight norms seem to hamper creativity (1, 3, 33, 34). Data from China’s rice areas found lower innovative thought style (2) and fewer patents for new inventions (17, 35). Rice farming and the tight norms associated with it seem to be mismatched with the skill set needed for innovation. However, rice-farming southern China may excel at *incremental* innovations, which are more common in tight societies (28).

In contrast, the more freewheeling wheat-farming areas of northern China are more likely to be hubs of innovation. China’s wheat areas are also probably easier places for newcomers to fit into, since loose societies are easier to acculturate to (37). These prior studies linking norm tightness to societal outcomes provide a road map of predictions that future studies can test across China.

### Will Rice–Wheat Differences Persist into the Future?

The analyses showed evidence for differences that fall along the historical borders of rice and wheat farming—a factor that was overlooked in earlier analyses (2). Overlooking the effect of historical subsistence styles is easy to do because they are no longer a part of most people’s everyday lives in China. For generations, most people in China worked in agriculture; only in 2003 did that number fall below 50% (38).

As more and more people enter apartment blocks and office jobs, the effects of China’s thousands of years of rice farming will become easier and easier to overlook. This gives researchers a unique opportunity. As it races to modernize, China gives researchers the chance to test in real time how culture changes.

Yet, the data here add to the evidence that rice culture is living on in modern China (19, 23), even among college students in big cities (17) and even among customers in Starbucks (39). Old patterns of rice farming are living on, at least for now. How our ancestors put food on the table has left a legacy on how we order society—not just in China, but around the world.

## Methods

We analyzed norm-tightness data from 11,662 participants using hierarchical linear models with respondents nested in provinces and in three survey waves (from ref. 2). Analyses took into account characteristics of respondents (gender and age) and provinces (log GDP per capita and urbanization). Because the urbanization ratio from the original paper was skewed (2.69), we used percentages. Percentages were less skewed (0.68) and predicted tightness (*r* = 0.56) slightly more strongly than ratios (*r* = 0.52).

To measure historical rice farming, we used the percent of paddy fields per cultivated land, as in prior research (17). To represent historical farming, we used the earliest provincial data we could find, from the 1996 *China Statistical Yearbook *(40). These data correlate highly with 1918 data available for a subset of 22 provinces (*r*[22] = 0.95, *P* < 0.001). Thus, the 1996 data seem to adequately represent historical patterns of rice farming.

We used data on paddy rice rather than rice output. This is because rice output also includes dryland rice. Dryland rice is less productive and grows without the irrigation systems that force farmers to coordinate their behavior (7).

## Supplementary Material

Supplementary File

## Data Availability

Data and analysis scripts are publicly available in the Open Science Framework (https://osf.io/q3pjf/).
