# Supplementary material for: Historically rice-farming societies have tighter social norms in China and worldwide
Source: Proc Natl Acad Sci U S A. 2020 Jul 30;117(33):19816–24. doi: 10.1073/pnas.1909909117 (PMC7443949; doi:10.1073/pnas.1909909117)
Supplement: Supplementary File [file pnas.1909909117.sapp.pdf]

Supplemental Materials

Contents

Supplemental Figures S1-S5
Supplemental Tables S1-S12
1. Correlation Between Higher Holistic Thought and Lower Innovative Thinking Style
    1.1 Mediation Analysis for Rice and Innovative Thinking Style
2. Rice Statistics
    2.1 Measuring Rice
    2.2 Do 1996 Statistics Represent Historical Rice Farming?
    2.3 Skewness
    2.4 Is There a Rice Tipping Point?
3. Environmental Rice Suitability
4. Interdependent Subsistence Style Index
    4.1 Calculating the Subsistence Index
    4.2 Is Rice a Better Predictor Than Herding?
5. Robustness to Alternative Analysis Methods
    5.1 Excluding Non-Han Provinces
    5.2 Alternative Nesting Methods
6. General Farming versus Rice
7. Urbanization
    7.1 Pulling Apart Urbanization from Wealth
8. Temperature
9. Herding Cultures in China
10. Percent Ethnic Han
11. Distance from Beijing
12. Distance to the Coast
13. Pathogen Prevalence
14. Population Density
15. Education
16. Alternative Measures of Modernization
17. Statistical Models
18. GDP Data
19. Thought Style Measures
20. Percent Muslim
21. Rice-Wheat Border Analysis
22. Analyzing Temperature and Latitude Within Rice and Wheat Regions
23. Tightness May Be Important for Acculturation
24. Ecological Threat Index
25. Rice Controlling for Collectivism
26. Percent College Graduates
27. Historical Warfare
27.1 External Conflict
    27.2 Internal Rebellions
    27.3 Herding Cultures as a Proxy for Warfare
    27.4 Land Occupied by Japan in WWII
28. Spatial Auto-Correlation: Are Provinces Similar Merely Because They're Neighbors?
    28.1 Moran's I
    28.2 Individual-Level Statistical Independence
    28.3 Rice-Wheat Border Differences

www.pnas.org/cgi/doi/10.1073/pnas.1909909117

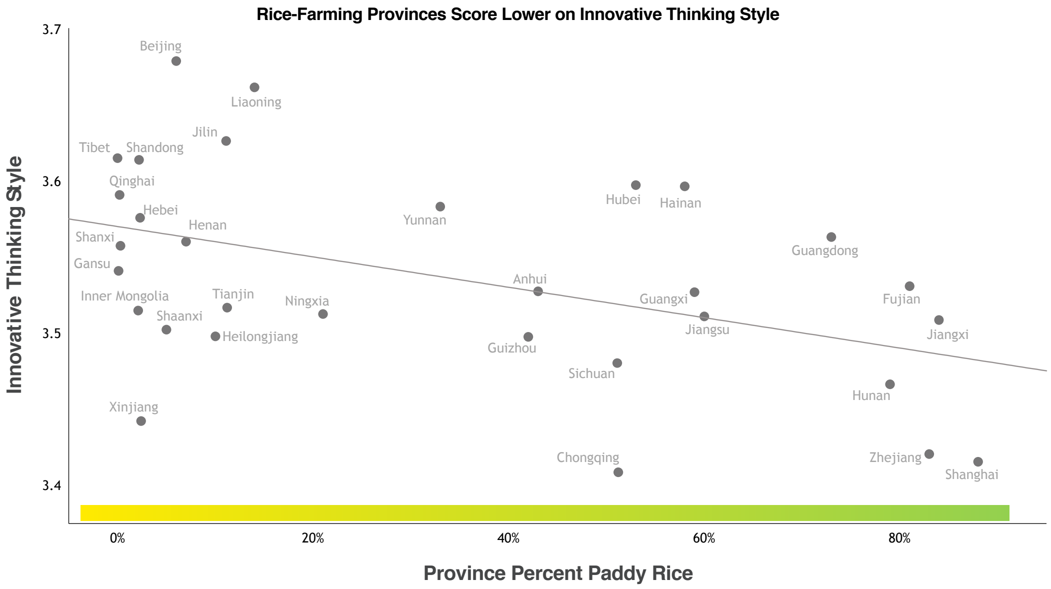

Figure S1. Rice provinces scored lower on Chua and colleagues' measure of innovative thinking style (Table 3).

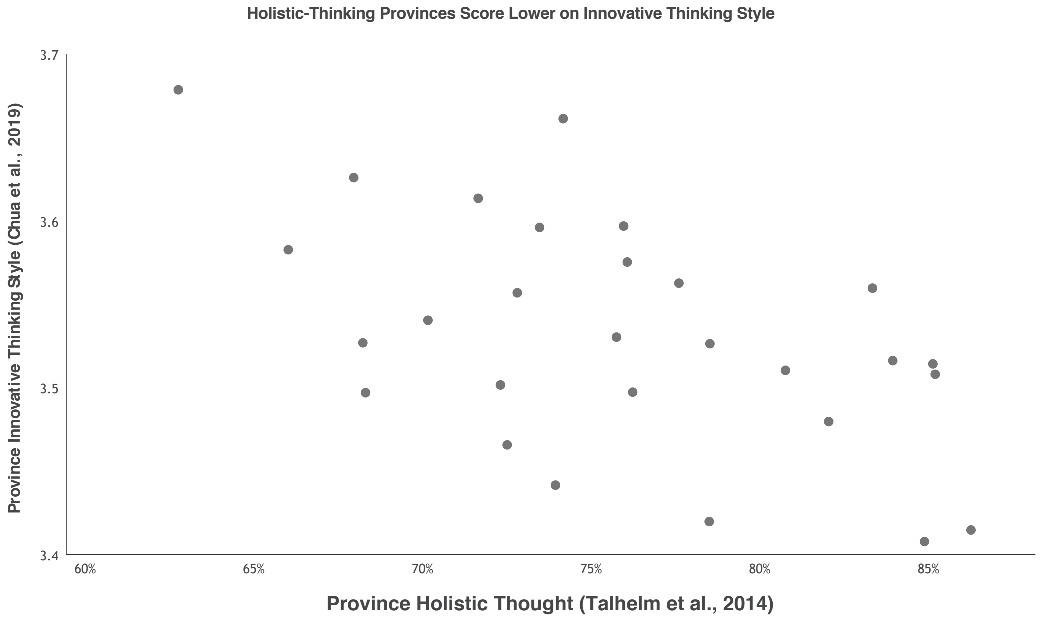

Figure S2. Provinces with more holistic thought tend to score lower on innovative thinking style, suggesting that the two measures tap into similar constructs. Each dot represents a province. Holistic thought is the percentage of relational pairings on the triad categorization task from Talhelm and colleagues (1). Innovative thinking style comes from a measure in the tightness-looseness study in China by Chua and colleagues (2). Rice significantly predicted innovative thinking style, but not the other two thought style sub-dimensions measured by Chua and colleagues (Table 3). Two outlying provinces with small samples that scored under 60% on holistic thought are not shown in the graph, but they are included in the analysis.

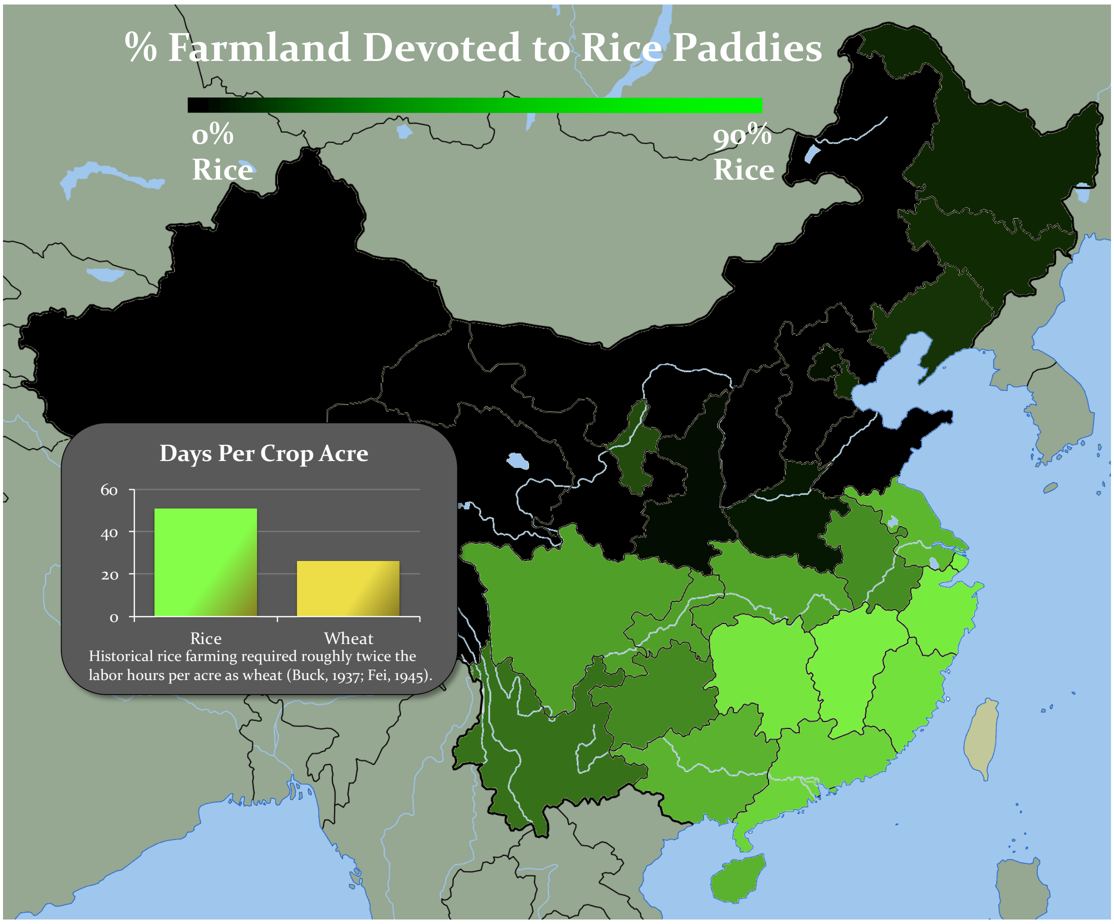

Figure S3. Paddy rice farming is centered in central China around the Yangtze River and further south. In the north, dryland crops like wheat, corn, and millet are common. Data comes from the 1996 *China Statistical Yearbook*.

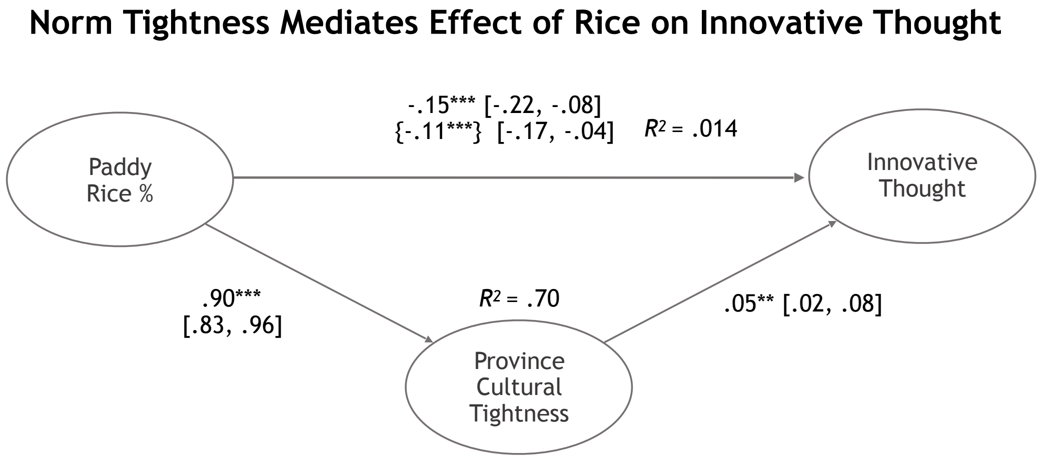

Figure S4. $*p < .05$; $**p < .01$; $***p < .001$; $N = 3,495$. Mediation analysis found that norm tightness partially mediated the link between rice and innovative thought. Values are regression coefficients [with bootstrapped 95% confidence intervals]. This mediation uses province-level cultural tightness. Figure S5 presents similar results using individual-level cultural tightness.

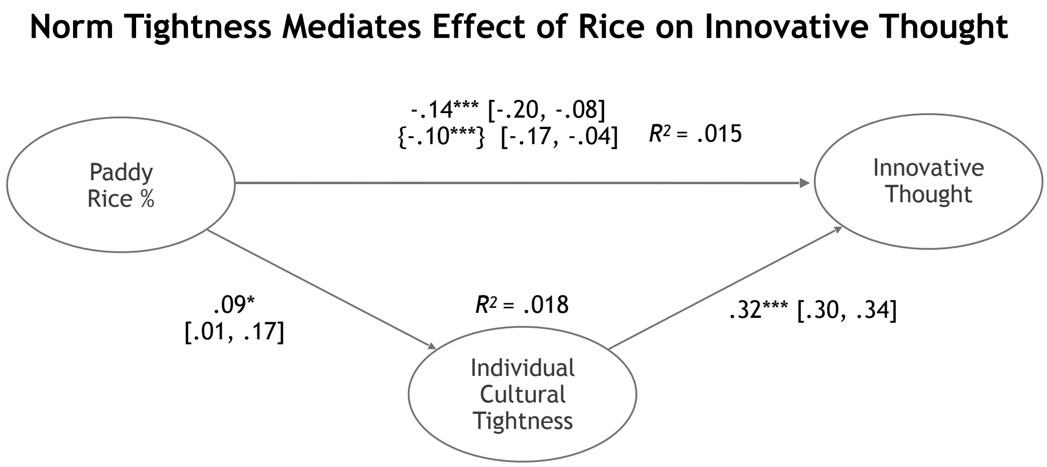

Figure S5. *p < .05; **p < .01; ***p < .001; N = 3,495. Mediation analysis found that norm tightness partially mediated the link between rice and innovative thought. Values are regression coefficients [with bootstrapped 95% confidence intervals]. This mediation uses individual-level cultural tightness. Figure S4 finds similar results using province-level cultural tightness.

Table S1: Part One

Variables Tested, Sources, and Rationale

Variable	Measure	Source	Rationale
China Regional Differences			
% Rice	Paddy fields area/total cultivated area	*China Statistical Yearbook*, 1996	Paddy rice required more work and coordination to build and operate irrigation systems.
Modern GDP	GDP per capita in 2008, 2012, 2015	*China Statistical Yearbook,* 2009, 2013, 2016	More developed provinces tend to have tighter norms (2).
Historical GDP	Log GDP Per Capita in 1995	*China Statistical Yearbook*, 1996	Studies have found a lag between economic growth and cultural change (3).
% Urban	Urban residents/total population, 2000, 2016	*China Population and Employment Statistical Yearbook*, 2001, 2017	Urbanized Chinese provinces tend to have tighter norms (2). We also test historical urbanization to test for a lagged effect.
% Cultivated Land	Hectares of cultivated land/total province land	*China Statistical Yearbook*, 1996	This measures the density of farming in general, pulling apart general farming and rice farming in particular.
Environmental Rice Suitability	Environmental suitability for high-input rainfed rice	UN Global Agro-Ecological Zones Database	Environmental suitability for rice (regardless of whether people actually farm rice there) helps address reverse-causality—the possibility that areas that were collectivistic to begin with chose to farm rice.
% Herding Cultures	People from herding ethnicities/total pop.	*China Pop. Stat. Yearbook*, 2002	Research has found that herding cultures tend to be more individualistic than nearby farming cultures (4).
Percent Han	People of Han ethnicity/ total province population	*China Population Statistical Yearbook*, 2002	The percent Han could be interpreted as a measure of ethnic homogeneity (lack of diversity) or as a proxy for Confucian heritage.
Distance from Beijing	Log distance of prov. capital city to Beijing (1,000 km)	Google Maps	Central government control may be stronger near the capital. Provinces nearer the central government have tighter norms on average (2).
Population Density	Population/province area	*China Stat. Yb*. 1996	Nations with higher population density tend to have tighter norms (5).
Historical Pop. Dens.	Population/province area	Shepherd, 1993	Historical population density predicts norms better than modern density (5).

Table S1: Part Two

Variables Tested, Sources, and Rationale

Variable	Measure	Source	Rationale
China Regional Differences			
Distance to Coast	Distance of prov. capital to nearest coast (100 km). Coast prov. = 0.	Marine Regions Database (from 6)	Distance from the coast can be a proxy for development (modern and historical). Coastal provinces also had more access to sea transport and potentially more diverse ideas and cultures.
Average Temperature	Average (average high, low in January and July)	Zuzu Che Weather Records	Some researchers have argued that hotter areas are more collectivistic (7). Temperature is correlated with disease prevalence (7).
Latitude	Average of northernmost and southernmost province latitude	Google Maps	In China, rice is highly correlated with latitude. Latitude is a proxy for other environmental factors such as temperature and disease. Testing latitude checks the robustness of rice against latitude.
Pathogen Prevalence	Average morbidity rates for human-transmitted diseases	*China Statistical Yearbook of Health*, 2001	Pathogen prevalence theory argues that environments with higher rates of communicable disease tend to be more collectivistic (8).
% College Graduates	College graduates per school-age population, 1990, 2015	*China Statistical Yearbook*, 1991, 2016	Researchers have argued that education is an important vehicle of modernization (9). We test modern and historical statistics.
% Service Industry	People employed in service jobs/employed people, 2010, 1995	*China Statistical Yearbook*, 2011, 1996	Some researchers have argued that service sector development is a better indicator of modernization than GDP (10). We test historical statistics because there is evidence for a lag between economic development and cultural change (3).
% Private Industry	People employed in priv. industry per employed people, 2010, 1995	*China Statistical Yearbook,* 2011, 1996	In China, the shift from the state-controlled economy to the private sector may be an indicator of modernization beyond GDP. We test historical statistics because there is evidence for a lag between economic development and cultural change (3).
Internet Penetration	Internet users/total province population	China Internet Development Report, 2008	Researchers have found some evidence that GDP statistics in China are sometimes manipulated (11). Internet installation rates are less politically sensitive and thus present an alternative indicator of modernization.
Qing Rebellion, Warfare	Frequency of rebellions, warfare in Qing Dynasty	Dincecco & Wang, 2018	Prior research has found tighter norms in places with a history of warfare (5). Beyond external warfare, rebellions killed millions in the Qing Dynasty (1644-1911).
Area Occupied by Japan in WWII	Proportion of provincial area occupied by Japan	Chua et al., 2019	Prior research has found tighter norms in places with a history of warfare (5), including this measure in China specifically (2).

Table S1: Part Three

Variables Tested, Sources, and Rationale

Variable	Measure	Source	Rationale
			Worldwide
% Rice Harvested Area	Irrig. wetland rice harv. area/cereal cultivation land in 2000	UNFAO & World Bank	Paddy rice required more work and coordination to build and operate irrigation systems.
Interdependent Subsistence Style Index	(Wheat harv. area/cereal cult. area) + (rice harv. area/cereal cult. area) – prop. pasture land.	Thomson et al., 2018	This index combines three subsistence styles linked to cultural differences: rice more interdependent than wheat (1), and herding which is more independent than farming in general (4).
GDP per Capita	GDP per capita, 2011 ($10,000, PPP)	World Bank	Economically developed areas have tighter norms in China (2), although this seems to not hold internationally (5).
Historical GDP	GDP per capita in 1950 ($1,000)	Maddison	Studies have found a lag between economic growth and cultural change (3).
Historical and Ecological Threats	Index of 7 threats, such as warfare, disease, and natural disaster	Gelfand et al., 2011; Thomson et al., 2018	Societies with histories of more ecological threats tend to have tighter norms (5). Threat index includes (i) history of territorial threats, (ii) demanding geoclimate, (iii) historical pathogen prevalence, (iv) tuberculosis incidence, (v) natural disaster vulnerability, (vi) population density in 1500, (vii) daily fat supply (reversed).
UN Human Development Index	Life expectancy, education, and income per capita, 2010	UN Human Development Report	The HDI is a broader index of development than GDP alone.
% Urban	Percent of people living in urban areas, 2018	CIA World Factbook	Studies have linked urbanization to differences in norm tightness (2, 12).

Table S2

Separating Urbanization and Wealth

	B/γ	SE	*t*	*P*
Male	0.001	0.012	0.10	0.924
Age	0.001	0.001	1.93	0.054
% Cultivated Land	0.11	0.11	0.97	0.336
% Rice	0.07	0.04	1.75	0.085
Log GDP per Capita	0.16	0.03	5.56	< 0.001
Male	0.0003	0.0124	0.02	0.984
Age	0.001	0.001	2.00	0.046
% Cultivated Land	0.25	0.12	2.02	0.047
% Rice	0.05	0.04	1.23	0.222
% Urban	0.40	0.13	2.99	0.004

Note: Table 1 has urbanization and wealth in the simultaneous regressions. Results here show that each on its own predicts tighter norms. But when put together (Table 1), urbanization predicts looser norms, in line with earlier results from the US (12). Analyses are hierarchical linear models with individuals nested in survey rounds nested in provinces. GDP data is log RMB from 2008. Urbanization is the percentage of urban residents per province in 2017.

Table S3A

Rice Farming Predicts Tighter Norms Controlling for Distance from Beijing and Distance to Coast

	B/γ	SE	*t*	*P*
Male	0.001	0.012	0.07	0.947
Age	0.001	0.001	1.89	0.058
GDP per Capita	0.33	0.06	5.76	< 0.001
% Urban	-0.86	0.26	-3.26	0.002
% Cultivated Land	0.14	0.11	1.31	0.193
% Rice	0.14	0.05	2.77	0.007
Distance from Beijing	-0.01	0.01	-0.80	0.429
Male	0.001	0.012	0.06	0.955
Age	0.001	0.001	1.90	0.058
GDP per Capita	0.32	0.06	5.49	< 0.001
% Urban	-0.76	0.24	-3.20	0.002
% Cultivated Land	0.13	0.12	1.06	0.293
% Rice	0.11	0.05	2.41	0.018
Distance to Coast	-0.001	0.003	-0.30	0.767

Note: Distance from Beijing is in 1,000 km, log transformed. Distance to Coast is in 100 km from the provincial capital, but zero for coastal provinces. Analyses are hierarchical linear models with individuals nested in survey rounds nested in provinces. GDP data is log RMB from 2008. Urbanization is the percentage of urban residents per province in 2017.

Table S3B

Temperature and Latitude Do Not Predict Tight Norms Within Rice and Wheat Regions, Where Rice and Temperature Are De-Confounded

Rice Region				
	B/γ	SE	*t*	*P*
Male	0.01	0.02	0.67	0.505
Age	0.002	0.001	1.65	0.099
GDP per Capita	0.35	0.13	2.69	0.043
% Urban	-0.63	0.69	-0.91	0.407
% Cultivated Land	-0.20	0.36	-0.56	0.602
Yearly Average Temperature (C)	-0.01	0.01	-0.42	0.677
Male	0.01	0.02	0.67	0.505
Age	0.002	0.001	1.65	0.099
GDP per Capita	0.36	0.13	2.77	0.040
% Urban	-0.67	0.68	-0.99	0.369
% Cultivated Land	-0.20	0.37	-0.54	0.614
Latitude	0.003	0.008	0.38	0.717
Wheat Region				
	B/γ	SE	*t*	*P*
Male	-0.01	0.02	-0.45	0.652
Age	0.003	0.001	2.80	0.005
GDP per Capita	0.26	0.08	3.22	0.007
% Urban	-0.64	0.30	-2.14	0.053
% Cultivated Land	0.31	0.15	2.05	0.063
Yearly Average Temperature (C)	-0.006	0.004	-1.36	0.199
Male	-0.01	0.02	-0.46	0.648
Age	0.003	0.001	2.81	0.005
GDP per Capita	0.28	0.09	3.03	0.010
% Urban	-0.70	0.31	-2.23	0.046
% Cultivated Land	0.25	0.16	1.61	0.133
Latitude	0.001	0.004	0.25	0.806

Note: Analyses on the left are within China's rice region (percent cultivated land ≥ 50% paddy rice); analyses on the right are within China's wheat region (< 50% rice). In China as a whole, rice is strongly correlated with temperature and latitude. But within the rice region and within the wheat region, there is very little variance in rice, but still large variation in temperature and latitude. Shaded rows correlate in the opposite direction from what theory would predict. GDP data is log RMB from 2008. Urbanization is the percentage of urban residents per province in 2017.

Table S4
Rice Farming Robust to Ethnic Han Makeup and Pathogen Prevalence

	B/γ	SE	*t*	*P*
Male	0.001	0.012	0.05	0.960
Age	0.001	0.001	1.90	0.058
GDP per Capita	0.34	0.06	5.62	< 0.001
% Urban	-0.85	0.26	-3.27	0.002
% Cultivated Land	0.09	0.13	0.71	0.478
% Rice	0.11	0.04	2.71	0.008
Percent Han	0.06	0.08	0.76	0.448
Male	0.001	0.012	0.06	0.954
Age	0.001	0.001	1.90	0.058
GDP per Capita	0.32	0.06	5.68	< 0.001
% Urban	-0.76	0.24	-3.24	0.002
% Cultivated Land	0.14	0.12	1.17	0.244
% Rice	0.11	0.04	2.86	0.005
Pathogen Prevalence	-0.0004	0.0036	-0.10	0.920

Note: GDP data is log RMB from 2008. Urbanization is the percentage of urban residents per province in 2017. Percent Han comes from the 2000 Census. Pathogen prevalence is a measure of morbidity from human-transmitted diseases per province (more details are in Supplemental Section 13).

Table S5

Population Density and Historical Urbanization

		B/γ	SE	*t*	*P*
Rice Robust to Population Historical Density	Male	0.001	0.012	0.05	0.958
	Age	0.001	0.001	1.90	0.058
	GDP per Capita	0.32	0.06	5.68	< 0.001
	% Cultivated Land	0.18	0.14	1.30	0.198
	% Rice	0.12	0.04	2.77	0.007
	% Urban 2017	-0.75	0.24	-3.15	0.002
	Population Density 1996	-0.21	0.54	-0.39	0.698
	Male	-0.003	0.014	-0.25	0.803
	Age	0.002	0.001	2.06	0.040
	GDP per Capita	0.38	0.07	5.26	< 0.001
	% Cultivated Land	0.02	0.19	0.11	0.916
	% Rice	0.11	0.05	2.22	0.031
	% Urban 2017	-0.93	0.29	-3.19	0.002
	Population Density in 1700s	-1.31	3.31	-0.39	0.695
Pop. Dens. W/o Urbanization	Male	0.001	0.012	0.10	0.920
	Age	0.001	0.001	1.87	0.061
	GDP per Capita	0.17	0.03	4.99	< 0.001
	Population Density 1996	0.25	0.41	0.61	0.545
	Male	-0.003	0.014	-0.20	0.840
	Age	0.002	0.001	2.01	0.044
	GDP per Capita	0.17	0.03	5.29	< 0.001
	Population Density in 1700s	0.93	2.41	0.39	0.702
Historical Pop. Dens.	Male	0.001	0.012	0.08	0.939
	Age	0.001	0.001	1.92	0.055
	GDP per Capita	0.24	0.06	4.32	< 0.001
	% Cultivated Land	0.08	0.11	0.66	0.509
	% Rice	0.07	0.04	1.90	0.061
	% Urban 2001	-0.27	0.16	-1.69	0.095

Note: Population density is a candidate for a mechanism between rice and norm tightness, but results find rice remains significant after taking into account both current and historical density. Rice becomes marginally significant using urbanization statistics from 2001, perhaps because pre-modern urbanization is a less effective predictor than modern urbanization. Population density 1996 is calculated as 100,000,000 people per km^2 from the 1996 *Statistical Yearbook*. Historical population density is calculated as 10,000 people per km^2 from *Statecraft and Political Economy on the Taiwan Frontier, 1600-1800*. GDP data is log RMB from 2008. Urbanization is the percentage of urban residents per province in 2001 (to represent pre-modern urbanization and 2017 to represent modern urbanization.

Table S6

Rice Farming is Robust to Individual Education and Regional Education Differences

	B/γ	SE	*t*	*P*	*B/γ*	SE	*t*	*P*	*B/γ*	SE	*t*	*P*
Male	0.004	0.015	0.25	0.802	0.001	0.012	0.07	0.944	0.001	0.012	0.07	0.943
Age	0.001	0.001	1.31	0.190	0.001	0.001	1.89	0.059	0.001	0.001	1.89	0.059
GDP per Capita	0.30	0.07	4.19	< 0.001	0.32	0.06	5.74	< 0.001	0.33	0.06	5.75	< 0.001
% Urban	-0.64	0.30	-2.14	0.037	-0.91	0.30	-2.99	0.004	-0.91	0.30	-3.02	0.003
% Cultivated Land	0.20	0.14	1.44	0.155	0.16	0.11	1.48	0.142	0.16	0.11	1.48	0.139
% Rice	0.12	0.05	2.47	0.017	0.13	0.05	2.89	0.005	0.13	0.05	2.92	0.004
Education Variable	0.01	0.01	1.38	0.168	0.002	0.003	0.74	0.462	0.01	0.01	0.78	0.437
	Respondent's Education				Province % College Grads 2015				Province % College Grads 1990			

Note: Respondents' education is coded from 1 to 5, with higher values representing more education (2). GDP data is log RMB from 2008. Urbanization is the percentage of urban residents per province in 2017.

Table S7A

Are Alternative Measures of Modernization Better Predictors of Tightness?

	B/γ	SE	*t*	*P*
Male	0.001	0.012	0.10	0.924
Age	0.001	0.001	1.87	0.061
GDP per Capita 2008	0.18	0.02	7.40	< 0.001
Male	-0.0002	0.0124	-0.02	0.984
Age	0.001	0.001	2.02	0.043
Log % Employed in Service Industry 2010	0.21	0.07	2.91	0.005
Male	0.001	0.012	0.07	0.942
Age	0.001	0.001	1.87	0.062
GDP per Capita 2008	0.21	0.03	6.66	< 0.001
Log % Employed in Service Industry 2010	-0.10	0.07	-1.34	0.183
Male	0.001	0.012	0.07	0.945
Age	0.001	0.001	1.89	0.059
GDP per Capita 2008	0.33	0.06	5.75	< 0.001
Log % Employed in Service Industry 2010	0.07	0.09	0.75	0.454
% Urban	-0.88	0.28	-3.12	0.003
% Cultivated Land	0.17	0.11	1.52	0.132
% Rice	0.12	0.04	2.99	0.004

Note: GDP per capita from 2008 is log-transformed RMB. Some researchers have argued that service sector development is a better indicator of modernization than GDP.

Table S7B

Are Alternative Measures of Modernization Better Predictors of Tightness?

	B/γ	SE	*t*	*P*
Male	0.001	0.012	0.05	0.960
Age	0.001	0.001	1.94	0.052
Log % Employed in Private Industry 2010	0.13	0.02	5.99	< 0.001
Male	0.001	0.012	0.10	0.922
Age	0.001	0.001	1.87	0.062
Log GDP per Capita 2008	0.17	0.05	3.61	< 0.001
Log % Employed in Private Industry 2010	0.01	0.04	0.26	0.799
Male	0.001	0.012	0.06	0.951
Age	0.001	0.001	1.89	0.059
Log GDP per Capita 2008	0.31	0.07	4.60	< 0.001
Log % Employed in Private Industry 2010	0.02	0.04	0.43	0.667
% Urban	-0.78	0.24	-3.27	0.002
% Cultivated Land	0.16	0.11	1.41	0.163
% Rice	0.11	0.04	2.65	0.010
Male	0.001	0.012	0.09	0.928
Age	0.001	0.001	1.99	0.047
Internet Penetration	0.85	0.12	7.00	< 0.001
Male	0.001	0.012	0.12	0.908
Age	0.001	0.001	1.90	0.057
Log GDP per Capita 2008	0.12	0.05	2.39	0.019
Internet Penetration	0.35	0.24	1.45	0.152
Male	0.001	0.012	0.08	0.939
Age	0.001	0.001	1.90	0.057
Log GDP per Capita 2008	0.24	0.07	3.39	0.001
Internet Penetration	0.54	0.26	2.04	0.044
% Urban	-0.86	0.23	-3.66	< 0.001
% Cultivated Land	0.23	0.11	2.04	0.044
% Rice	0.09	0.04	2.09	0.039

Note: Internet penetration statistics come from 2007 and represent an alternative measure of development from GDP statistics, which some have questioned as manipulated. In China, the shift from the state-controlled economy to the private sector may also be an indicator of modernization beyond GDP. GDP per capita from 2008 is log-transformed RMB.

Table S8A

Are Historical Measures of Modernization Better Predictors of Tight Norms?

	B/γ	SE	*t*	*P*
Male	0.001	0.012	0.10	0.924
Age	0.001	0.001	1.87	0.061
Log GDP per Capita 2008	0.18	0.02	7.40	< 0.001
Male	0.002	0.013	0.16	0.873
Age	0.001	0.001	1.55	0.121
% Employed in Service Industry 1995	0.010	0.002	4.74	< 0.001
Male	0.003	0.013	0.22	0.829
Age	0.001	0.001	1.43	0.152
Log GDP per Capita 2008	0.19	0.04	4.86	< 0.001
% Employed in Service Industry 1995	-0.001	0.003	-0.43	0.667
Male	0.003	0.013	0.21	0.831
Age	0.001	0.001	1.48	0.139
Log GDP per Capita 2008	0.33	0.06	5.60	< 0.001
% Employed in Service Industry 1995	0.009	0.004	2.44	0.017
% Urban	-1.28	0.32	-4.01	< 0.001
% Cultivated Land	0.25	0.11	2.17	0.033
% Rice	0.14	0.04	3.53	< 0.001

Note: GDP statistics are log transformed. Urbanization is the percentage of urban residents per province in 2017. Service sector and private employment are alternative measures of modernization. These statistics from the 1990s represent time-lagged, historical measures of modernization.

Table S8B

Are Historical Measures of Modernization Better Predictors of Tight Norms?

	B/γ	SE	*t*	*P*
Male	0.001	0.013	0.11	0.912
Age	0.001	0.001	1.58	0.114
% Employed in Private Industry 1995	4.00	0.95	4.21	< 0.001
Male	0.003	0.013	0.22	0.824
Age	0.001	0.001	1.43	0.152
Log GDP per Capita 2008	0.19	0.03	5.33	< 0.001
% Employed in Private Industry 1995	-0.27	1.15	-0.23	0.816
Male	0.002	0.013	0.19	0.852
Age	0.001	0.001	1.48	0.138
Log GDP per Capita 2008	0.34	0.06	5.41	< 0.001
% Employed in Private Industry 1995	-1.11	1.23	-0.91	0.368
% Urban	-0.74	0.25	-2.93	0.004
% Cultivated Land	0.15	0.11	1.38	0.170
% Rice	0.13	0.04	2.97	0.004
Male	0.001	0.012	0.09	0.928
Age	0.001	0.001	1.91	0.056
Log GDP per Capita 1995	0.18	0.02	7.61	< 0.001
Male	0.001	0.012	0.10	0.920
Age	0.001	0.001	1.89	0.059
Log GDP per Capita 2008	0.07	0.07	0.96	0.338
Log GDP per Capita 1995	0.12	0.07	1.70	0.092
Male	0.001	0.012	0.06	0.954
Age	0.001	0.001	1.87	0.061
Log GDP per Capita 2008	0.22	0.08	2.69	0.009
Log GDP per Capita 1995	0.13	0.08	1.70	0.092
% Urban	-0.84	0.24	-3.57	< 0.001
% Cultivated Land	0.14	0.11	1.31	0.193
% Rice	0.09	0.04	2.04	0.045

Note: GDP statistics are log transformed. Urbanization is the percentage of urban residents per province in 2017. Private employment is an alternative measure of modernization. These statistics from the 1990s represent time-lagged, historical measures of modernization.

Table S8C

Are Alternative Measures of Modernization Better Predictors of Tightness?

	B/γ	SE	*t*	*P*	*B/γ*	SE	*t*	*P*	*B/γ*	SE	*t*	*P*
Male	0.001	0.012	0.09	0.928	0.001	0.012	0.05	0.960	0.001	0.012	0.05	0.960
Age	0.001	0.001	1.90	0.057	0.001	0.001	1.94	0.052	0.001	0.001	1.94	0.052
GDP per Capita	0.005	0.001	6.50	< 0.001	0.004	0.001	5.46	< 0.001	0.004	0.001	5.52	< 0.001
Male	0.001	0.012	0.10	0.924	0.001	0.012	0.05	0.958	0.001	0.012	0.06	0.956
Age	0.001	0.001	1.87	0.061	0.001	0.001	1.94	0.053	0.001	0.001	1.94	0.053
Log GDP per Capita	0.18	0.02	7.40	< 0.001	0.19	0.03	6.03	< 0.001	0.20	0.03	6.12	< 0.001
	GDP per Capita 2008				GDP per Capita 2012				GDP per Capita 2015			

Note: Time-lagged GDP per capita is a stronger predictor (with larger *t* values) of social norms than the concurrent GDP statistics from 2015 (used in the original study). Log GDP (bottom) tends to predict more strongly than regular GDP (top).

Table S9

Rice-Wheat Differences Are Robust to Alternative Nesting and Excluding non-Han Provinces

Nested in Provinces (Survey Fixed Effects)				
	B/γ	SE	*t*	*P*
Male	0.0004	0.0125	0.03	0.975
Age	0.002	0.001	2.25	0.024
Survey Round 2	0.18	0.02	12.10	< 0.001
Survey Round 3	0.16	0.01	10.80	< 0.001
GDP per Capita	0.34	0.07	5.08	< 0.001
% Urban	-0.80	0.28	-2.92	0.008
% Cultivated Land	0.15	0.13	1.20	0.243
% Rice	0.12	0.05	2.47	0.022
Excluding Non-Han Provinces				
	B/γ	SE	*t*	*P*
Male	-0.004	0.013	-0.29	0.772
Age	0.002	0.001	2.43	0.015
Survey Round 2	0.18	0.22	0.82	0.414
Survey Round 3	0.15	0.22	0.71	0.480
GDP per Capita	0.39	0.07	5.53	< 0.001
% Urban	-0.96	0.29	-3.31	0.001
% Cultivated Land	0.01	0.12	0.12	0.905
% Rice	0.10	0.04	2.45	0.017

Note: GDP per capita is 2008 log-transformed RMB. Urbanization is the percentage of urban residents per province in 2017. Survey rounds are dummy coded with Round 1 as the reference category.

Table S10

Rice Predicts Tighter Norms Across Nations Beyond GDP, Development, and Historical GDP

Norm Tightness Around the World				
	B/γ	SE	*t*	*P*
% Rice Harvested Area	5.71	1.69	3.38	0.002
Log 2011 GDP per Capita	-0.82	0.65	-1.26	0.218
% Rice Harvested Area	5.18	1.63	3.17	0.004
UN Human Development Index	-7.42	3.75	-1.98	0.058
% Rice Harvested Area	4.61	1.85	2.49	0.019
1950 GDP per Capita	-0.29	0.21	-1.40	0.173

Note: Rice statistics represent the percentage of cereal production area harvested with rice in the year 2000. GDP per capita from 2011 is in units of $10,000 international dollars log transformed, accounting for purchasing power parity. The 1950 GDP per capita data is in units of $1,000. The Human Development Index data incorporates health, education, and economic data from 2010. Tightness-looseness scores come from Gelfand and colleagues' study of 32 nations (5).

Table S11

Subsistence Styles Predict Tight Norms Across Societies Beyond Modern GDP, Historical GDP, and Development

Norm Tightness Around the World				
	B/γ	SE	*t*	*P*
Interdependent Subsistence Style Index	4.20	1.56	2.70	0.011
Log 2011 GDP per Capita	-0.46	0.69	-0.67	0.510
Interdependent Subsistence Style Index	3.75	1.53	2.45	0.020
UN Human Development Index	-6.59	4.04	-1.63	0.113
Interdependent Subsistence Style Index	3.79	1.82	2.08	0.047
1950 GDP per Capita	-0.28	0.22	-1.24	0.224

Note: Rice statistics represent the percentage of cereal production area harvested with rice in the year 2000. GDP per capita from 2011 is in units of $10,000 international dollars, accounting for purchasing power parity. The 1950 GDP per capita data is in units of $1,000. The Human Development Index data incorporates health, education, and economic data from 2010. Tightness-looseness scores come from Gelfand and colleagues' study of 32 nations (5).

Table S12

Rice and Subsistence Styles Predict Tight Norms in China and Worldwide Beyond Differences in Collectivism

China				
	B/γ	SE	*t*	*P*
Male	0.03	0.02	1.42	0.156
Age	0.003	0.001	1.91	0.056
GDP per Capita	0.27	0.07	3.60	0.001
% Urban	-0.007	0.003	-2.39	0.025
% Rice	0.19	0.05	3.63	0.001
In-Group Collectivism	0.14	0.01	12.62	< 0.001
Male	0.04	0.02	1.54	0.124
Age	0.001	0.001	0.89	0.372
GDP per Capita	0.27	0.08	3.62	0.001
% Urban	-0.007	0.003	-2.13	0.043
% Rice	0.18	0.05	3.24	0.003
Relational Collectivism	0.04	0.01	4.09	< 0.001
Worldwide				
	B/γ	SE	*t*	*P*
% Rice Harvested Area	4.64	1.46	3.19	0.004
Ingroup Collectivism	1.11	0.54	2.08	0.049
% Rice Harvested Area	4.74	2.01	2.36	0.025
Individualism (Hofstede)	-0.02	0.02	-0.77	0.446
Interdependent Subsistence Style Index	4.01	1.40	2.86	0.009
Ingroup Collectivism	1.34	0.54	2.48	0.021
Interdependent Subsistence Style Index	3.52	1.60	2.20	0.036
Individualism (Hofstede)	-0.03	0.02	-1.45	0.158

Note: We encourage readers to interpret these analyses carefully, because collectivism is also a plausible consequence of rice and the subsistence style index. These analyses suggest that rice may cause differences in norm tightness beyond the effects of collectivism, at least as collectivism is measured in these survey items. In our prior writings, we have raised the possibility that survey items measuring collectivism may not capture the type of tight, duty-bound culture of rice villages (13, 14). Collectivism values in China come from survey items administered in the earlier study on norm tightness (2). International collectivism data come from the GLOBE study (15), and individualism values come from Hofstede's international studies of individualism (16). China GDP data is log RMB from 2008. Urbanization in China is the percentage of urban residents per province in 2017. Worldwide Rice statistics represent the percentage of cereal production area harvested with rice in the year 2000.

1. Correlation Between Higher Holistic Thought and Lower Innovative Thinking Style

Our prior study found higher holistic thought in China's rice provinces, as well as lower rates of patents for inventions (1). Other studies have found that people who think analytically (often contrasted with holistic thought) tend to score higher on measures of creativity (17). As a test of convergent validity, we tested whether our earlier province-level scores of holistic thought (1) predicted people's individual-level scores of innovative thinking style from the *PNAS* study (2).

Chua and colleagues used a different measure of thought style—innovative thought style. Is innovative thought style related to holistic thought style? To investigate this idea, we tested whether our earlier provincial scores for holistic thought across China (1) were correlated with individuals' innovative thinking style. Provincial holistic thought scores did predict lower innovative thought style $\gamma = -0.41$, $P = 0.009$, $r_{prov} = -0.66$ (Fig. S3). This suggests that holistic thought is tapping into something similar to low innovative thought style.

This evidence suggests a link between holistic thought, lower creativity, and lower rates of patents for inventions. Figure S2 illustrates this relationship with province-level means for holistic thought and innovative thought style. (Supplemental Section 19 tests whether there is a significant amount of variation in thought style between provinces.)

The findings on innovative thought style here may fit with earlier findings on holistic versus analytic thought. Researchers have contrasted analytic thought style with holistic thought, which relies more on intuition and experience than formal logic (18). Studies have found that analytic thinkers tend to score higher on measures of creativity (17). In line with this theory, the measure of innovative thinking from Chua and colleagues was negatively correlated with our

prior data on holistic thought across China (Fig. S3). In short, holistic-thinking provinces also tended to score lower on innovative thinking.

1.1 Mediation Analysis for Rice and Innovative Thinking Style

We analyzed mediation between rice and innovative thought using the PROCESS Model 4 with 5,000 bootstrap iterations (19). We report mediation tests of tightness both at the province level and the individual level. However, we argue that the province-level test is more conceptually sensical than the individual-level test. This is because (a) researchers think of tightness as a characteristic of cultures more so than individual people and (b) using provincial tightness scores avoids the problem of correlating two self-report variables from the same person. Two self-report variables from the same participants can be correlated for reasons other than a true relationship. For example, patterns of how different participants tend to answer survey questions can inflate correlations between variables.

As in the main analyses, we controlled for age, gender, GDP 2008, the percent of cultivated land, and urbanization. The mediation was significant (B = 0.043 [95% CI = .013; .072], $Z = 2.81$, $P = .005$, Fig. S5). At the individual level, the mediation was significant but smaller (B =.029 [95% CI = .001; .051], $Z = 2.31$, $P = .020$, Fig. S6). In short, the mediation analysis suggested that the link between rice farming and lower innovative thought style can be partially explained by norm tightness. However, the mediation effect was partial, suggesting there are other mechanisms besides tightness.

2. Rice Statistics

2.1 Measuring Rice

To measure rice farming, we used the percentage of cultivated land devoted to paddy fields from 1996, the earliest *Statistical Yearbook* we could locate. Note that paddy rice does not

include dryland rice. Some varieties of rice can grow on dry land. However, dryland rice is far less productive and likely less important for culture because its labor demands are lower and lacks irrigation networks that tie farmers together (13).

2.2 Do 1996 Statistics Represent Historical Rice Farming?

In the main text, we ask whether these modern statistics represent historical farming. Fortunately, one source reports rice acreage and total grain acreage for 22 provinces from 1914-1918 (20). The high correlation ($r[20] = 0.95$, $P < 0.001$) suggests that the 1996 data adequately represents historical rice farming.

2.3 Skewness

We analyzed the skewness and kurtosis of the rice data around the world. Both were under the recommended cutoffs (21). Skewness was 1.80 (versus cutoff of 2) and kurtosis was 1.89 (versus cutoff of 7). However, some readers may wonder whether 1.80 is still too close to the recommended cutoff. Thus, we re-ran the main rice analysis in Table 2 using square-root transformed rice data.

Square-root transformation reduced skewness to 1.21. Repeating the top analysis in Table 2, square-root-transformed rice continued to predict norm tightness (ß = 0.56, $P = .001$). This relationship was slightly stronger than for untransformed rice (ß = 0.52, $P = .003$).

2.4 Is There a Rice Tipping Point?

One unanswered question in research on rice farming is whether there is a tipping point at which rice starts to influence culture. In the main text, we use the percentage of rice farming as a continuous variable, but here we test two alternative methods to model the effect of rice on culture:

1. Rice squared

Rice squared can test whether there's an additive effect of rice—where the effects on culture are particularly acute as nearly all the farmland is rice.

2. A categorical rice variable

For a categorical variable, we argue that a reasonable value to test is a 50% cutoff. This could be a tipping point, since 50% is when rice makes up the majority crop and could plausibly take on the dominant role in the local culture.

We compared the province-level effect size of (1) the full continuous rice variable versus (2) rice squared, and (3) a 50% categorical variable (rice above or below 50% of crop land). The full continuous variable had a marginally smaller effect size (r_{prov} = .20) than rice squared (r_{prov} = .27) and the categorical variable (r_{prov} = .27). This result lends some support to the idea that differences on the low end of rice percentages are not as important for influencing culture. However, the sample size is limited to 31 provinces, which makes it hard parse out the difference between three variations of the same variable. This possibility of a tipping point is worth testing in future studies across different outcomes and particularly in county-level data, which would allow for a more detailed test.

3. Environmental Rice Suitability

Environmental rice suitability came from the United Nations Food and Agriculture Organization's Global Agro-Ecological Zones database. The database provides a suitability index for high-input rainfed rice per cultivated land in each province. Importantly, this index does not take into account whether people are farming rice on the land or not. Rice suitability strongly predicted actual rice. Some researchers have suggested that instrumental variables should predict the key variable with an *F* statistic of 10 or larger (22). The *F* statistic for

environmental suitability predicting actual rice farming (88.01) far surpassed this cutoff, suggesting that environmental suitability is a suitably strong instrumental variable.

This data can get help get at the question of self-selection. If paddy rice were suitable all over China but only grown in certain areas, that would suggest self-selection. For example, perhaps areas with tighter norms were more likely to start farming rice. However, the United Nations data showed that paddy rice is suitable mostly along the Yangtze River and further south.

The results in the main text find that environmental rice suitability strongly predicts actual rice farming. This result makes sense with crop data from China finding that rice yielded five times more per hectare than wheat (13, 23). Rice was hard work, but it paid off. This suggests that the environment largely determined where rice is farmed in China. It is inconsistent with the hypothesis that most of China could farm rice, but only certain areas self-selected into farming rice.

4. Interdependent Subsistence Style Index

4.1 Calculating the Subsistence Index

To calculate the interdependent subsistence style index, we followed the prior study on relational mobility across countries that used this index (24). This index combines the three subsistence styles that have received the most research in relation to individualism: wheat, rice, and herding. It is based on the idea that research has uncovered a continuum in interdependence from herding (least interdependent) to settled agriculture (more interdependent) to rice farming (13).

However, this index is new and unrefined. It is unrefined partly because many other common subsistence styles have not been researched in detail. For example, few studies have

analyzed fishing and corn farming, even though these are major subsistence styles. In contrast, there is a small but building base of evidence finding that farming cultures tend to be more interdependent than herding cultures and hunter-gatherers (4, 25, 26). There is also mounting evidence that rice is a particularly interdependent form of farming (1, 6, 13, 27). Thus, this index is rooted in the latest subsistence style research, but we expect it to be refined in the future.

To calculate the index, we used international wheat and rice statistics from the United Nations Food and Agriculture Organization's Global Agro-Ecological Zone database. This database reports the number of hectares harvested with wheat and wetland rice in the year 2000. The wheat statistic includes both rainfed and irrigated wheat. This is then turned into a percentage of the number of hectares under cereal production in the year from the World Bank.

Herding was measured based on data from the United Nations Food and Agricultural Organization's Corporate Statistical Database. The data reports land devoted to pasturing in different countries. This dataset puts countries into five bins. To convert the categories into numbers, we used the midpoint of the range for each category. This data is from 1990—the earliest data available from this source.

It is important to remember that we are trying to estimate the historical subsistence tradition of different cultures. This is particularly difficult in the case of settlement cultures like Singapore and Hong Kong. Singapore and Hong Kong have little or no farming or herding on their land. Yet the land of these city-states does not represent the long-term cultural heritage of these populations. Thus, to remedy this, we used data from the areas that were the source of the majority ethnic Han Chinese settlers to these areas. More details on these measures and estimating the subsistence history of settlement populations are explained in Supplemental Section 1.5 in the prior study on relational mobility (24).

Because herding data was unavailable for Belgium, we imputed the value using data from neighboring France. Belgium did have data in the year 2000, at which point its pasture data was equal to France. Thus, the 1990 France data seems to be a reasonable proxy, especially given that more than 50% of the land area of Belgium is French-speaking.

In addition, the pasture data from China required special consideration. The pasture statistic for China almost certainly overestimates the importance of herding in China historically. This is because China has a large landmass, with vast herding areas that have only a tiny percentage of the population. In large landmasses with highly uneven population distribution, a population-weighted statistic would be more appropriate. Because the vast majority of Han Chinese live in farming areas more akin to the land-use makeup of South Korea, Japan, and India, we imputed the pasture figure from India (these three countries are binned in the same category in the data). In contrast to China, India does not have the same extent of vast, sparsely populated herding areas. Thus, the statistic from India likely better represents the experience of the vast majority of the Han Chinese population.

4.2 Is Rice a Better Predictor Than Herding?

We report in the main text that both rice and herding predict tightness on their own. Rice is a slightly stronger predictor ß = 0.51, P = .003 than herding ß = -0.45, P = .010. One possibility is that rice is simply confounded. (In other words, nations that farm rice tend not to herd.) They are correlated $r(30) = -.49$, P = .005. However, only about 25% of the variance is overlapping (R squared = .24).

That raises the question of what happens when rice and herding are in the same model. When put together, rice emerges as the stronger predictor ß = 0.39, P = .037. Herding still predicts less tight norms, but it's marginally significant ß = -0.26, P = .148.

Thus, perhaps we throw out herding then? We think herding should still be included. For one, there is reason from prior research to think it should matter (28). Second, although it's not significant, adding herding does modestly improve model fit. Rice alone explains 26.3% of the international variance in norm tightness, but adding in herding increases that to 31.5%.

These results might lead to the conclusion that the subsistence style index should be tweaked. We created the index as a simple, direct measure—wheat, herding, and rice. There's no weighting or more complicated modeling. We designed it this way to make the index transparent to readers and to avoid overfitting the index to a single dataset.

However, it is plausible that the different subsistence styles should be weighted differently. For example, since the effect of rice is stronger than herding, perhaps it should be weighted higher. As a simple test of this idea, we computed a modified subsistence style index where rice was double weighted. This index outperformed (R squared = 28.5%) the simple index (R squared = 20.9%).

Despite this model improvement, we hesitate to change the main analyses to the modified index. It's possible this updated index is really just overfitting to a single dataset. Instead, we think it's prudent to refine the model over time as more studies are done and the model can be fitted across different outcome measures.

5. Robustness to Alternative Analysis Methods

5.1 Excluding Non-Han Provinces

In prior studies, we have presented analyses excluding the major non-Han provinces of Tibet, Xinjiang, and Inner Mongolia. Because these provinces have different ethnic makeups, religions, and languages, they may confound the comparison between rice and wheat regions.

Thus, tested the robustness of the results to excluding these provinces. Analyses found that rice continued to predict norm tightness after excluding these provinces (Table S9).

5.2 Alternative Nesting Methods

To take into account the three different rounds of the survey, we ran the main analyses with participants nested in provinces, nested in survey rounds. However, to test the robustness of the analysis to nesting, we ran alternative analyses with main effects of survey rounds and participants nested only in provinces (Table S9). We also ran simple regressions (non-hierarchical). Rice remained significant across these different analysis methods.

6. General Farming versus Rice

To measure the density of farming in general—rather than rice specifically—we used statistics on the percentage of cultivated land per province from the same *Statistical Yearbook* as the rice data, 1996. Because Chongqing was still a part of Sichuan province in 1996, we assigned it the value of Sichuan. Cultivated land is highest around north central China, in provinces such as Shandong, Henan, and Jiangsu. Fortunately for the purposes of pulling general farming and rice farming apart, rice is not confounded with farming density in China, as measured by the percentage of cultivated land per province, $r(29) = 0.042$, $P = 0.823$.

7. Urbanization

As a measure of urbanization, we used statistics on the percentage of urban residents per province from the 2017 *China Population and Employment Statistical Yearbook* (中国人口和就业统计年鉴), as in our prior study (29). As a test of more historical urbanization, we used year 2000 statistics from the *China Population Statistical Yearbook 2001* (中国人口统计年鉴).

The original paper used a ratio of urban to rural population from 2015, rather than the percentage of urban population (2). However, our analyses of this ratio found that its skewness (2.69) was above the recommended cutoff of two (21). In contrast, the percentage data we use is under the cutoff for both the modern data (0.68) and historical data (1.44). The skewness of the ratio data might explain why the percentage data is a slightly stronger predictor of tightness (r_{prov} = 0.56 versus 0.52). Thus, we use the percentage statistic rather than the ratio statistic.

Historical urbanization was not a stronger predictor than modern urbanization (Table S5). In the model with historical urbanization, rice became borderline significant ($P = 0.061$). This could be because historical urbanization was a weaker predictor and thus the model was doing an inferior job of taking into account differences in norm tightness that are due to urbanization (which is modestly correlated with rice, $r[29] = 0.31$, $P = 0.155$). In line with this explanation, rice became significant again after adding in modern urbanization statistics ($P = 0.004$).

7.1 Pulling Apart Urbanization from Wealth

The main text present analyses that pull apart effects of GDP and urbanization. However, it is worth pointing out that GDP and urbanization are highly correlated. Because these two variables are so highly correlated, a more definitive test would be to run county-level analyses. A county-level analysis would allow for a more precise test of urbanization and development.

8. Temperature

As a measure of temperature, we used the average yearly temperature in Celsius of the capital city of each province. We calculated the average of the average high and low in the coldest month (January) and hottest month (July) from http://weather.zuzuche.com/c280.html. Supplemental Section 22 reports tests of temperature and latitude within the rice-wheat regions, which helps de-confound these variables from rice.

9. Herding Cultures in China

As a proxy for historical herding, we used the percentage of traditional herding ethnicities from each province according to the 2000 Fifth National Census (reported in the *China Population Statistical Yearbook 2002*). The judgment of which groups practiced a meaningful amount of herding historically is fairly easy for China's largest herding groups: the Uyghurs, Tibetans, Mongolians, Manchus, and other Turkic groups such as the Kyrgyz. This becomes more difficult for smaller groups, about which less is written, such as the Daur and Luoba. However, because these groups make up such a small portion of the population, disagreements about their subsistence style end up making little difference in the final numbers. The following is the full list of herding groups: Uyghur, Mongolians, Manchu, Tibetan, Kyrgyz, Salar, Daur, Xibo, Tajik, Uzbek, Ewenke, Yugur, Tatar, Elunchun, Hezhe, Menba, Luoba, and Kazakh.

10. Percent Ethnic Han

We gathered the percentage of ethnic Han per province from the same yearbook as the herding data. Percent Han could be interpreted as a measure of ethnic homogeneity (or, in other words, the lack of diversity). It could also be considered a proxy for Chinese culture, such as Confucian heritage. Percent Han did not predict regional differences in tightness-looseness.

11. Distance from Beijing

We calculated the distance from Beijing using an online map and the capital city of each province. China's provincial capitals tend to be located in the center of each province. We calculated this in units of 1,000 kilometers. We log transformed distance based on the idea that the distance between, say, 1 and 200 kilometers may be more meaningful in the human mind than the difference between 2,001 and 2,200 kilometers. We added 1 to all values in order to

avoid taking a log of zero (for Beijing). Regardless of transformation, distance from Beijing did not significantly predict tightness after taking into account GDP and urbanization.

12. Distance to the Coast

We used data on distance from the coast data from the Marine Regions database (used in 6). This data takes the distance from the central point of a region to the nearest coast. Distance to the coast was zero for all coastal provinces. The units are in hundreds of kilometers.

Distance to the coast may be important for several reasons. For one, coastal provinces tend to be more developed. Coastal provinces also had more access to sea transport and the activities that go along with it, such as trade and invasion.

13. Pathogen Prevalence

One theory of cultural differences in collectivism is pathogen prevalence theory (8). Pathogen prevalence researchers argue that areas in environments with higher rates of communicable disease tend to be more collectivistic. Collectivism is relevant to norm tightness because collectivistic cultures tend to have tighter norms (Table S2 from 5). The theory is that if there are lots of pathogens in an environment, strangers are more likely to be carrying dangerous diseases. In response, cultures become more insular and wary of outsiders.

To measure pathogen prevalence, we used average morbidity rates for human-transmitted diseases from the 2001 *China Statistical Yearbook of Health*. We used morbidity rates rather than mortality rates because (a) mortality was far rarer, which raises concerns about whether the measurement is robust, and (b) humans are presumably attuned to serious diseases, even in the cases where people don't die from the disease. The yearbook reports rates for 26 communicable diseases, including plague, cholera, hepatitis (type A, B, C, and E), dysentery, typhoid fever, HIV, gonorrhea, syphilis, TB, malaria, and others. China classifies these 26 diseases as "A or B

class infectious diseases" (甲、乙类法定传染病). We focused on the primarily human-transmitted disease because pathogen prevalence researchers have found that human-transmitted diseases are stronger predictors (30). Thus, we excluded four diseases that the Ministry of Agriculture reports infects both domestic animals and humans, such as rabies.

14. Population Density

As a measure of population density, we used total population per province from the 1996 *Statistical Yearbook* divided by the area of the province. Note that Chongqing was not a province then, so we imputed data from the 1999 *China Statistical Yearbook* (which reports 1998 statistics), the earliest available on the statistical bureau's website. Population density did not significantly predict differences in tightness (Table S5).

We also tested historical population density from the 1700s. This data comes from *Statecraft and Political Economy on the Taiwan Frontier, 1600-1800*. This is correlated with rice, but weakly $r(20) = .17, p = 0.455$. Like with modern population density, historical density is much more correlated with the percentage of cultivated land $r(20) = .76, p < 0.001$. For example, that dense wheat province (Shandong) had 127 people per square kilometer compared to just 72 in Guangdong (a major rice province).

Unfortunately for the task of pulling apart different causal factors, historical density has the same confounds as modern density. Historical density is highly correlated with urbanization ($r[20] = .59, p = 0.004$) and GDP ($r[20] = .67, p = 0.001$). Like modern population density, historical density correlates with norm tightness ($r[20] = .58, p = 0.005$) but falls away after controlling for GDP ($t = 0.39, p = 0.702$; Table S5). Thus, historical density leads to similar conclusions as modern density.

Population density from the 1700s uses historical political divisions and so does not map perfectly onto modern provinces. Thus, we used nearby geographic regions to increase the sample size. Because Chongqing was a part of Sichuan Province until 1997, we assigned it the value for Sichuan. Because Shanghai previously belonged to Jiangsu Province, we assigned Shanghai with Jiangsu's number. The historic province of Chihli (*zhili,* 直隶) has no direct analogue today, but is in the area of the modern-day Beijing-Tianjin-Hebei corridor, which we assigned numbers for. Obviously these imputations reduce some of the specificity of the data, but they provide reasonable proxies that allow for a larger sample size (22 provinces).

15. Education

To measure regional differences in education, we collected statistics on the percentage of college graduates per province. The statistical yearbook reports the population as the population over age six, which is the age of schooling. However, for calculating the portion of college graduates, age six is not the ideal age cutoff, since six-year-olds don't go to college. For contrast, the US Census uses age 25 and above. The closest age categorization we could find in the statistical yearbook was age 15 and above. Thus, we used the percentage of college graduates in the population aged 15 and above.

Some modernization researchers have argued that education is a particularly important vehicle of modernization (9). In addition, many people in China link education (教育) to concepts like “refinement” (修养). For example, when considering “loose” behaviors like spitting in public and talking loudly on a cell phone, it is fairly common for people to explain these behaviors as a lack of education and refinement.

We tested both modern (year 2015) and historical (1990) statistics on the percentage of college graduates per province. This variable ranges widely between provinces. For example, in

the 2015 data, Henan province had 10.1% college graduates, whereas Beijing had 44.4%. Even among provinces that are not major cities, variation is still high. For example, Liaoning province has nearly double (18.2%) the college graduates as Henan.

In Table S6, we also test education at the individual level. Rice remained significant after considering individual-level education, regional education, and historical education. Participants' education did not significantly predict norm tightness. Because the original study only measured education during two data waves, we did not include individual-level education as a control variable in other analyses. We chose this analysis method because (a) individual-level education did not significantly predict tightness and (b) including education would have required excluding thousands of participants.

16. Alternative Measures of Modernization

Modernization researcher Ronald Inglehart argued that GDP is not always the best indicator of modernization (10). Other China-watchers have argued that GDP statistics are unreliable because they are tied to promotions for regional leaders (11). For that reason, in this section, we test whether alternative indicators are a better predictor of tightness than GDP. For each indicator, we test modern and historical indicators, because there is some evidence that historical indicators like GDP and population density are a better predictor of modern-day cultural differences (5, 24).

The analyses find that GDP per capita remains the best predictor either when compared in separate models or in simultaneous models (Tables S7-S8). Thus, the main analyses retain GDP per capita. However, the results find that rice continues to predict tightness after adding these alternative indicators of modernization. Below we describe each indicator in detail.

Service Sector. One modernization variable that Inglehart points to is the development of the service sector (10). While GDP represents the size of the economy, the service sector may better reflect the shift toward more modern service sector positions, such as accountants, tour guides, and flight attendants. The world wealthiest countries also tend to have a high percentage of service sector employment, but there are exceptions. For example, countries that sit on mining or gas resources can get a large GDP without a modern economy.

As a measure of the service sector, we analyzed statistics on the percentage of employed people in service jobs per province from the *China Statistical Yearbook*. In order to test both recent and historical indicators, we collected data from the 1996 and 2010 yearbooks. For the 2010 data, we searched for more recent data to match the year of the survey, but we were unable to find employment data broken down into different sectors in later years.

Since Inglehart has argued that service sector development may be a better indicator of modernization, we tested whether it predicted tightness better than GDP. Results in Tables S7A and S8A find that service sector development (modern and historical) was not a stronger predictor of tightness than GDP.

Private Industry. In China, the switch from a state-owned economy to the private sector is another strong candidate for an indicator of modernization. To test this, we collected statistics on the percentage of employed people who are employed in private industry per province from the 2011 *China Statistical Yearbook*. As with service sector employment, we could not find statistics later than 2010, so we used 2010 to represent more recent statistics and 1996 data to represent more historical development.

Results for private industry were similar to results for service sector development. For both historical and modern data, private industry development did not predict tightness more

strongly than GDP per capita (Tables S7B and S8B). Thus, privatization did not seem to be a better indicator of modernization in the case of norm tightness.

Internet Penetration. Finally, we analyzed internet penetration statistics because there is some evidence that GDP statistics are influenced by local leaders (11). The idea is that local leaders have an inventive to inflate GDP statistics because their promotions depend on economic growth. To get around this shortcoming, we analyzed statistics on internet penetration, which are presumably less sensitive to faking.

We collected internet penetration statistics from the 2008 *China Internet Development Report*. Internet penetration rates varied widely, from 6% in Guizhou province to 47% in Beijing. We did not collect later statistics because internet penetration was quite high in later years, with less meaningful variation. As with the other modernization statistics, we found no evidence that internet penetration statistics were better predictors than GDP (Table S7).

17. Statistical Models

We ran hierarchical linear models using the LMER function in the statistical program R. We ran models with provinces nested in survey rounds with the following code format:

```
lmer(tightness ~ Predictors +(1|Round/Province))
```

In the Table S9, we test the robustness of this analysis method against alternative forms of nesting the data and to non-hierarchical models. Rice remains a significant predictor across these different forms of analysis.

18. GDP Data

The original paper used 2015 GDP per capita. Because GDP is often not best fit as a linear predictor, we tested whether log GDP per capita was a stronger predictor. Results showed

that log GDP produced larger *t* values (t = 6.12 versus 5.52; although both were significant, and the difference in regression coefficients was not significant).

Other research has found a lag between economic growth and culture change (3, 24). This suggests that using GDP statistics of the same year the survey was conducted may not be optimal. Consistent with this idea, we tested concurrent (2015), 2012, and 2008 GDP statistics (Table S8C). This analysis found that year 2008 log GDP per capita was a stronger predictor than 2015 log GDP per capita (t = 7.40 versus 6.12; although both were significant). Thus, we used log-transformed GDP statistics from 2008.

Would going farther back into history produce a stronger predictor? Tables S8A and S8B present analyses of GDP and alternative measures of modernization going back to 1995. The alternative predictors were weaker than 2008 GDP. However, 1995 log GDP per capita was a close contender. In individual regressions, its *t* value was slightly higher than 2008 (t = 7.40 versus 7.61). However, in a simultaneous model, 2008 GDP remained significant (t = 2.69), while 1995 GDP did not (t = 1.70). The main analyses use log-transformed 2008 GDP per capita (tests of alternatives GDP and modernization statistics are in Tables S7 and S8).

19. Thought Style Measures

Chua and colleagues used a thought style measure developed by Kirton (31). The scale has 32 items and starts with the following instructions: "Imagine that you had been asked to present, consistently and for a long time, a certain image of yourself to others. Please indicate the degree of difficulty that such a task would entail…" Items include "Has original ideas," "Never acts without proper authority," and "Is methodical and systematic."

The analysis correlating holistic thought and innovative thought style in the main text uses individual-level innovative thought style scores, but Figure S2 aggregates innovative

thought style scores to the province level. Does it make sense to aggregate Chua and colleagues' thought style to province-level averages? We ran a one-way ANOVA to test whether there was a significant amount of variation between provinces in innovative thought style. Results found that province membership accounted for a significant amount of variation in innovative thought style $F(30, 3464) = 1.77$, $P = 0.006$.

20. Percent Muslim

Because several rice cultures are predominately Muslim cultures (such as Pakistan), we tested whether rice farming was robust to Islam. We collected the percentage of the Muslims in the population of different countries. The CIA World Factbook reports this statistic for many countries around the world. Where this was unavailable, we used statistics from Pew Research.

Controlling for the percentage of the population that is Muslim, rice continued to predict tightness ß = 0.45, $P = 0.001$. Because many rice cultures are in Asia, we added an "Asia" dummy variable to the model to test whether rice was robust to simply being in the Asian continent. In this model, rice remained significant ß = 0.57, $P = 0.018$. These results suggest that rice is robust to relationships with Islam and the Asian continent. Another way to shed light on this question would be to collect tightness-looseness data in more non-Asian rice-farming areas, such as parts of West Africa that farm rice.

21. Rice-Wheat Border Analysis

In Tables 1, S3, and S4, we analyze climate and geographic variables to separate rice from other geographical differences. Here we use another method to pull apart rice from geographic factors like temperature. This analysis takes advantage of the fact that the distribution of rice is different from the distribution of factors like temperature.

To do this, we compared all neighboring provinces along China's rice-wheat border. These provinces were Qinghai, Gansu, Shaanxi, Henan, Anhui, Shandong (< 50% rice), Jiangsu, Hubei, Chongqing, and Sichuan (> 50% rice). All of these are neighboring provinces. For China as a whole, wheat areas and rice areas differ 12.5 degrees in latitude. Along the rice-wheat border, the difference is 4.0 degrees. For China as a whole, wheat areas and rice areas have a difference of 8.6 degrees Celsius in yearly average temperature. Along the rice-wheat border, the difference is 4.8 degrees Celsius.

Table 1 reports the results of the rice-wheat border analysis. The percentage of rice farming per province continued to predict differences in norm tightness along the rice-wheat border. Although analyzing neighboring areas cannot definitively prove that rice is a cause of culture and not a confound of temperature, these analyses suggest that rice-wheat differences persist, even when differences in factors like latitude and temperature are minor.

Note that this analysis is different from an analysis in our earlier study (1). In that study, we compared nearby *counties* in the same provinces along the rice-wheat border. Unfortunately, Chua and colleagues' data does not include city-level data. Thus, we were limited to comparing neighboring provinces, rather counties.

22. Analyzing Temperature and Latitude Within Rice and Wheat Regions

Pulling apart rice, temperature, and latitude is difficult in China. Across Chinese provinces as a whole, rice is strongly correlated with temperature $r(29) = 0.78$, $P < 0.001$ and latitude $r(29) = -0.74$, $P < 0.001$. Rice and temperature are so strongly correlated that it makes little sense to control for one or the other.

However, there are ways around this problem. Table 1 shows the results of one method—comparing neighboring provinces along the rice-wheat border. Another way around this problem

is to test whether temperature predicts cultural differences *within* the rice region and *within* the wheat region.

Within the wheat region (< 50% cultivated land devoted to rice paddies), there is little variation in rice, but still large variation in temperature and latitude. Even limiting the comparison to wheat provinces, yearly average temperature still varies from 2.3 to 18.0 degrees Celsius. As a comparison, that's roughly the difference between Sweden and Syria. Latitude also ranges widely, from 25 degrees to 48 degrees north. That's equivalent to moving from Egypt to Germany.

In the rice region (> 50% cultivated land devoted to rice paddies), temperature ranges from 15.8 to 24.0 Celsius. That's roughly similar to the difference between Greece and the Congo. Meanwhile, latitude ranges from 19.2 to 33.0 degrees north—roughly the span from Haiti to Oklahoma. In sum, analyzing within the rice and wheat regions allows us to minimize differences in rice and wheat while retaining large variation in temperature and latitude.

The results of analyses within the rice and wheat regions show no support for effects of temperature or latitude (Table S3B). Of course, because the number of provinces is limited (rice = 12 provinces; wheat = 19 provinces), it is not surprising that temperature and latitude were not significant in any of the four analyses (Ps ≥ 0.199). However, it is revealing that the correlation is in the wrong theoretical direction in every analysis. For example, if higher temperature caused tighter norms, temperature should be positively correlated with norm tightness. Yet within each region, temperature was weakly *negatively* correlated with norm tightness.

Latitude was also correlated in the wrong theoretical direction. If northern latitudes led to weaker norms, latitude should be negatively correlated with norm tightness. Yet within each region, latitude was weakly *positively* correlated with norm tightness.

The results of these analyses are consistent with the idea that (a) temperature and latitude do not drive differences in norm tightness and (b) rice-wheat differences across China are not likely to be confounds of temperature and latitude. When considered along with evidence from natural experiments like an isolated rice-farming county in northern China (27), the weight of the evidence suggests that rice-wheat differences are independent from temperature and latitude.

There are other ways to get at this question. For example, studies have been able to draw stronger conclusions by testing in places like India, where rice and temperature are naturally unrelated (32) and by testing at the county level in China, which gives far more degrees of freedom (6).

23. Tightness May Be Important for Acculturation

These differences in tightness around China may be important for acculturation. A study of tightness around the world found that people who moved from tight cultures to loose cultures adapted better than people who moved from loose cultures to tight cultures (33). If this pattern holds in China, it suggests that it may be easier for people to adapt to China’s wheat-farming areas. This has practical implications for the millions of Chinese people who move around the country for work and study. This idea is worth testing in future studies.

24. Ecological Threat Index

Gelfand's research has found that countries that experienced ecological threats tend to have tighter norms (5). The theory is that human societies use tight norms to help cope with threats like war and disease. In contrast, when times are plentiful and peaceful, societies can afford to have looser norms.

To measure threats, we used a composite index of seven threats from a study on relational mobility around the world (24). The index is based on the earlier study of norm tightness around

the world (5). This index includes: (i) history of territorial threats (warfare), (ii) climatic demands, (iii) historical pathogen prevalence, (iv) modern disease as indexed by incidence of tuberculosis per 100,000 people, (v) natural disaster vulnerability, (vi) historical population density from the year 1500, and (vii) fat supply per day (reverse coded to represent food scarcity). Details and sources can be found in the international study of relational mobility (24).

25. Rice Controlling for Collectivism

An anonymous reviewer requested that we run analyses of rice controlling for collectivism. We hesitate to do this because collectivism is a plausible consequence of rice farming (1), which would mean it is potentially illogical to control for collectivism. However, results show that rice continued to predict tighter norms within China and around the world, even after taking into account differences in collectivism (Table S12).

If rice causes collectivism, and collectivism is linked to tighter norms (Table S2, 5), this result is a puzzle. Why would rice still be linked to tight norms after accounting for collectivism? There are a few plausible explanations.

1. Rice is linked to more than collectivism. For example, there's evidence that rice-farming cultures have lower relational mobility (24), and studies have found that relational mobility mediates cultural differences (24, 34). For example, one study found that differences in relational mobility could explain why Americans share more personal details than people in Japan (34). In sum, collectivism is not the only plausible consequence of rice.

2. Even though collectivism is linked to tight norms, the relationship is far from perfect. Across 26 nations, collectivism and tightness correlate at $r(24) = .49$ (Table S2, 5). Gelfand and colleagues argue that collectivism and norm tightness are "related but distinct constructs" (5).

Thus, even if we assume collectivism causes norm tightness, the size of the correlation would mean that we should only expect collectivism to explain about 25% of the variance in tightness.

3. Even if we assume that rice causes collectivism, which causes tightness, these self-report survey scales are imperfect measures of collectivism. Multiple studies have documented trouble measuring collectivism across cultures (35–38). For example, researchers tested people in the US, UK, Germany, and Japan on multiple measures of social style and cognitive style (38). On the (non-self-report) behavioral measures, Americans scored more individualistic than the Europeans, who were in turn more individualistic than participants from Japan. Yet on self-report scales, participants from the US scored the *highest* on collectivism, while people in Japan scored the lowest. This was not a one-off finding. A meta-analysis of 76 cross-cultural studies found that the odds that self-report scales found that places like China or Japan scored higher on collectivism than North Americans was no different from flipping a coin (36). Among all the self-report items, this could be a particular problem for rice-wheat differences when scale items assess feelings of trust or intimacy with other people, which are paradoxically *lower* in societies with low relational mobility (24).

As a measure of collectivism in China, we used the survey items from Chua and colleagues' paper on norm tightness in China (2). They measured group collectivism and relational using items from the GLOBE survey (15). For example, one group collectivism item asked participants to rate their agreement with the statement, "In this society, being accepted by the other members of a group is very important." ("在本省市，被某一群体 的其他成员接受十分重要.") One relational collectivism item read, "In this society, children take pride in the

individual accomplishments of their parents." ("儿童会因为自己父母的 个人成就而感到自豪
").

As a measure of collectivism across nations, we used the nation scores for in-group collectivism from the GLOBE survey (15). Scores were available for both collectivism and tightness for 26 countries. We also tested individualism scores from Hofstede, which were available for 32 countries that also had tightness scores (16).

26. Percent College Graduates

To test whether education differences could explain regional differences in norm tightness in China, we gathered data on the percentage of college graduates per province. We divided the number of college graduates by the school-age population (which is age 6 and above in China). We gathered statistics representing modern (2015) and historical (1990) data from the *China Statistical Yearbook*, 1991, 2016.

27. Historical Warfare

The original study on norm tightness around the world found that societies that experienced more frequent warfare in the past have tighter norms (Table S3, 5). Thus, we tested whether norms were tighter in provinces that experienced more historical warfare.

27.1 External Conflict

First, we analyzed data on the number of battles in wars with external enemies during the Qing Dynasty (39). To be sure, Chinese history extends far beyond the Qing Dynasty (1644-1911), and warfare from before the 1600s could plausibly have an influence on regional culture. However, it is most plausible that (all else equal) more recent historical events would generally have a stronger influence on culture than events farther back in history.

The 300 years of the Qing Dynasty is certainly not a complete view of Chinese history. A full study could be devoted to the question of how to model warfare in Chinese history. But the Qing Dynasty is a plausible place to start in the analysis of historical warfare's influence on society.

As a measure of warfare, we averaged the battle geodata Dincecco and Wang on external warfare to the province level (39). Based on a simple correlation at the province level, warfare was a borderline significant predictor of tight norms $r(29) = 0.36$, $P = 0.050$. However, after including other predictors, warfare became non-significant (Table 4). This seemed to be because warfare was modestly more common in wealthier provinces $r(29) = 0.32$, $P = 0.079$. Warfare became non-significant after adding GDP to the model. Meanwhile, rice remained significant after taking warfare into account.

27.2 Internal Rebellions

There is a case to be made that internal uprisings were more important than external conflicts in the last 300 years. A fair amount of historical warfare in China was between herding groups and the predominately farming Han. Researchers who tallied historical conflicts in China found that, "more than 80% of external conflicts in China between 1000 and 1799 were fought against the nomads" (39).

However, the Qing Dynasty was set up by the herding Manchus. Thus, they were no longer a threat. Furthermore, the Qing Dynasty expanded so far that the old border of the Great Wall was no longer significant. Meanwhile, internal rebellions were common—and devastating (40). For example, the Taiping Rebellion is estimated to have killed 10-30 million people. Thus, in the 400 years of Chinese history encompassed by the Qing Dynasty, rebellions and other internal conflict may have affected more people than external warfare. We tested internal

rebellion using data on the frequency of mass rebellions during the Qing Dynasty from Wang (40).

The rebellion data told a different story from the warfare data. Rebellions were more common in provinces that are now more economically developed $r(29) = 0.51$, $P = 0.003$ (GDP per capita). Based on the simple correlation at the province level, norms were slightly stronger in areas with more rebellions, although it was not significant $r(29) = 0.26$, $P = 0.167$. But after taking economic development into account, rebellions predicted marginally *less* tight norms (Table 4). Rice remained significant after controlling for rebellions.

27.3 Herding Cultures as a Proxy for Warfare

Because so many conflicts in China were with herding cultures (39), the percentage of herding cultures in a region could be a proxy for the frequency of warfare. Of course, this measure is not perfect because warfare is not confined to groups' home region—especially with nomadic groups! However, this metric should function as a rough proxy. In line with this idea, the percentage of herding peoples correlated with the frequency of external conflict in the Qing Dynasty $r(29) = 0.48$, $P = 0.007$. The percentage of herding peoples could be an indicator of warfare that stretches farther back into history beyond the Qing Dynasty (39).

Rice remained a significant predictor of norm tightness after taking herding peoples into account (Table 1). To the extent that herding is a proxy for historical warfare, this result suggests that rice-wheat differences are not a confound of warfare. In sum, rice remained significant across three different methods for accounting for historical warfare. Although no single measure is a perfect index of historical warfare, the results across multiple indicators suggest that rice is robust to accounting for historical warfare.

27.4 Land Occupied by Japan in WWII

We also tested data on the proportion of land in each province occupied by Japan in WWII from the study of Chua and colleagues (2). This data did not include Inner Mongolia. Occupation was more extensive in China's wealthy coastal provinces (see note at Table 4).

28. Spatial Auto-Correlation: Are Provinces Similar Merely Because They're Neighbors?

When comparing differences across geography, it is important to consider the possibility that places might be similar to each other merely because they are physically close to each other. Proximity matters. Cultural diffusion is real.

However, cultural diffusion is not monolithic. Some phenomena are clustered than others. If spatial auto-correlation (clustering) is high, we might find a spurious relationship with other geographic features that are clustered, such as rice, which is naturally clustered because of climate.

To address this question, we present several factors:

28.1 Moran's I

We estimated the extent of spatial auto-correlation in norm tightness in China using Moran's I (I = 0.27). As with correlations, Moran's I values range from -1 to 1.

How big is this? We interpret this as relatively low spatial auto-correlation compared to other group-level social phenomena. Here are a few examples for comparison.

- County per-capita income in a study of one US state found I = 0.38.
- County-level voting results in the 2008 US presidential election were auto-correlated at I = 0.58.
- Around the world, the syntax of where languages place adverbs in sentences is I = 0.63.

Thus, group-level auto-correlation of norm tightness in China seems to be modest.

28.2 Individual-Level Statistical Independence

Another issue with geographic data is that people are not independent observations. Instead, people are clustered by geography. Thus, two participants from, say, Henan province should not be treated as statistically independent.

The main analyses in the paper (such as Table 1) are hierarchical linear model with participants clustered in provinces. This method takes into account the fact that individuals are clustered and not independent.

Geographic space is not the only form of clustering. Participants in this dataset are also clustered in survey rounds (which could have effects such as time of the year or recency of news events that could affect people's attitudes). Thus, we also cluster analyses within survey rounds and test survey fixed effects in Table S9. Rice remains robust in these analyses that take into account clustering.

28.3 Rice-Wheat Border Differences

Finally, the border analysis can give some insight into spatial clustering. If spatial clustering were a strong determinant of norm tightness, we would expect the neighboring provinces along the rice-wheat border to be similar to each other. That would work against the hypothesis of rice-wheat differences. If spatial clustering or (diffusion by mere proximity) were strong, we would expect differences to be smaller at the border. However, the differences in norm tightness were just as large among these neighboring provinces ($r_{\text{prov}} = 0.43$) as for China as a whole ($r_{\text{prov}} = 0.33$).

In sum, there is evidence for spatial clustering of norm tightness in provinces in China. However, clustering is relatively modest (I = 0.27). In addition, comparisons of provinces that

are close to each other but differ in rice and wheat suggest that rice-wheat differences are independent of spatial clustering.

Supplemental References

1. T. Talhelm, *et al.*, Large-scale psychological differences within China explained by rice versus wheat agriculture. *Science* **344**, 603–608 (2014).

2. R. Y. Chua, K. G. Huang, M. Jin, Mapping cultural tightness and its links to innovation, urbanization, and happiness across 31 provinces in China. *Proc. Natl. Acad. Sci.*, 201815723 (2019).

3. I. Grossmann, M. E. Varnum, Social structure, infectious diseases, disasters, secularism, and cultural change in America. *Psychol. Sci.* **26**, 311–324 (2015).

4. A. K. Uskul, S. Kitayama, R. E. Nisbett, Ecocultural basis of cognition: Farmers and fishermen are more holistic than herders. *Proc. Natl. Acad. Sci.* **105**, 8552–8556 (2008).

5. M. J. Gelfand, *et al.*, Differences between tight and loose cultures: A 33-nation study. *Science* **332**, 1100–1104 (2011).

6. J. Zhu, J. B. Ang, P. G. Fredriksson, The agricultural roots of Chinese innovation performance. *Eur. Econ. Rev.* **118**, 126–147 (2019).

7. V. Guernier, M. E. Hochberg, J.-F. Guégan, Ecology drives the worldwide distribution of human diseases. *PLoS Biol.* **2**, e141 (2004).

8. C. L. Fincher, R. Thornhill, D. R. Murray, M. Schaller, Pathogen prevalence predicts human cross-cultural variability in individualism/collectivism. *Proc. R. Soc. Lond. B Biol. Sci.* **275**, 1279–1285 (2008).

9. A. Inkeles, D. Smith, *Becoming modern: Individual changes in six developing societies* (Harvard University Press, 1974).

10. R. Inglehart, P. Norris, *Rising tide: Gender equality and cultural change around the world* (Cambridge University Press, 2003).

11. J. L. Wallace, Juking the stats? Authoritarian information problems in China. *Br. J. Polit. Sci.* **46**, 11–29 (2016).

12. J. R. Harrington, M. J. Gelfand, Tightness–looseness across the 50 united states. *Proc. Natl. Acad. Sci.* **111**, 7990–7995 (2014).

13. T. Talhelm, S. Oishi, “How rice farming shaped culture in Southern China” in *Socioeconomic Environment and Human Psychology*, A. K. Uskul, S. Oishi, Eds. (Oxford University Press, 2018), pp. 53–76.

14. S. Liu, M. W. Morris, T. Talhelm, Q. Yang, Ingroup vigilance in collectivistic culture. *Proc. Natl. Acad. Sci.* **116**, 14538–14546 (2019).

15. M. J. Gelfand, D. Bhawak, L. Nishii, D. J. Bechtold, "Individualism and collectivism" in *Culture, Leadership and Organizations: The GLOBE Study of 62 Societies*, R. J. House, P. J. Hanges, M. Javidan, P. W. Dorfman, V. Gupta, Eds. (Sage, 2004), pp. 473–512.

16. G. Hofstede, Values Survey Module (2013) (December 2, 2018).

17. H. A. Witkin, C. A. Moore, D. R. Goodenough, P. W. Cox, Field-dependent and field-independent cognitive styles and their educational implications. *Rev. Educ. Res.* **47**, 1–64 (1977).

18. R. E. Nisbett, K. Peng, I. Choi, A. Norenzayan, Culture and systems of thought: Holistic versus analytic cognition. *Psychol. Rev.* **108**, 291–310 (2001).

19. A. F. Hayes, *Introduction to mediation, moderation, and conditional process analysis: A regression-based approach* (Guilford Publications, 2017).

20. J. L. Buck, *Land utilization in China* (Chicago University Press, 1935) (July 31, 2017).

21. P. J. Curran, S. G. West, J. F. Finch, The robustness of test statistics to nonnormality and specification error in confirmatory factor analysis. *Psychol. Methods* **1**, 16–29 (1996).

22. D. O. Staiger, J. H. Stock, Instrumental variables regression with weak instruments. **65**, 557–586 (1997).

23. D. H. Perkins, *Agricultural development in China, 1368-1968* (Aldine, 1969).

24. R. Thomson, *et al.*, Relational mobility predicts social behaviors in 39 countries and is tied to historical farming and threat. *Proc. Natl. Acad. Sci.* **115**, 7521–7526 (2018).

25. J. W. Berry, Independence and conformity in subsistence-level societies. *J. Pers. Soc. Psychol.* **7**, 415–418 (1967).

26. H. Barry, I. L. Child, M. K. Bacon, Relation of child training to subsistence economy. *Am. Anthropol.* **61**, 51–63 (1959).

27. X. Dong, T. Talhelm, X. Ren, Teens in rice county are more interdependent and think more holistically than nearby wheat county. *Soc. Psychol. Personal. Sci.* **10**, 966–976 (2018).

28. A. K. Uskul, H. Over, Responses to social exclusion in cultural context: Evidence from farming and herding communities. *J. Pers. Soc. Psychol.* **106**, 752–771 (2014).

29. T. Talhelm, A. Kathmandu, X. Wang, Rice-Wheat Thought Style Differences Emerge by Age 10 and Widen Through Age 15 (in prep).

30. R. Thornhill, C. L. Fincher, D. R. Murray, M. Schaller, Zoonotic and non-zoonotic diseases in relation to human personality and societal values: Support for the parasite-stress model. *Evol. Psychol.* **8**, 151–169 (2010).

31. M. Kirton, Adaptors and innovators: A description and measure. *J. Appl. Psychol.* **61**, 622–629 (1976).

32. T. Talhelm, The rice theory of culture (doctoral dissertation) (2015).

33. N. Geeraert, R. Li, C. Ward, M. J. Gelfand, K. A. Demes, A tight spot: How personality moderates the impact of social norms on sojourner adaptation. *Psychol. Sci.* **30**, 333–342 (2019).

34. J. Schug, M. Yuki, W. Maddux, Relational mobility explains between- and within-culture differences in self-disclosure to close friends. *Psychol. Sci.* **21**, 1471–1478 (2010).

35. K. Peng, R. E. Nisbett, N. Y. Wong, Validity problems comparing values across cultures and possible solutions. *Psychol. Methods* **2**, 329–344 (1997).

36. S. J. Heine, D. R. Lehman, K. Peng, J. Greenholtz, What's wrong with cross-cultural comparisons of subjective Likert scales? The reference-group effect. *J. Pers. Soc. Psychol.* **82**, 903–918 (2002).

37. S. J. Heine, E. E. Buchtel, A. Norenzayan, What do cross-national comparisons of personality traits tell us? The case of conscientiousness. *Psychol. Sci.* **19**, 309–313 (2008).

38. S. Kitayama, H. Park, A. T. Sevincer, M. Karasawa, A. K. Uskul, A cultural task analysis of implicit independence: Comparing North America, Western Europe, and East Asia. *J. Pers. Soc. Psychol.* **97**, 236–255 (2009).

39. M. Dincecco, Y. Wang, Violent conflict and political development over the long run: China versus Europe. *Annu. Rev. Polit. Sci.* **21**, 341–358 (2018).

40. Y. Wang, "For Whom the Bell Tolls: The Political Legacy of China's Cultural Revolution" (Working Paper, 2019).
